# Receptor kinase FERONIA regulates flowering time in *Arabidopsis*

**DOI:** 10.1186/s12870-019-2223-y

**Published:** 2020-01-16

**Authors:** Long Wang, Tao Yang, Qinlu Lin, Bingqian Wang, Xu Li, Sheng Luan, Feng Yu

**Affiliations:** 1 College of Biology, State Key Laboratory of Chemo/Biosensing and Chemometrics, and Hunan Province Key Laboratory of Plant Functional Genomics and Developmental Regulation, Hunan University, Changsha, 410082 People’s Republic of China; 2 National Engineering Laboratory for Rice and By-product Deep Processing, Central South University of Forestry and Technology, Changsha, 410004 People’s Republic of China; 3 National Key Laboratory of Plant Molecular Genetics, Chinese Academy of Sciences Center for Excellence in Molecular Plant Sciences, Institute of Plant Physiology and Ecology, Shanghai Institutes for Biological Sciences, Chinese Academy of Sciences, Shanghai, 200032 People’s Republic of China; 4 Department of Plant and Microbial Biology, University of California, Berkeley, CA 94720 USA

**Keywords:** FERONIA, Circadian clock, Flowering, mRNA alternative splicing, RALF1 peptide

## Abstract

**Background:**

The receptor-like kinase FEROINA (FER) plays a crucial role in controlling plant vegetative growth partially by sensing the rapid alkalinization factor (RALF) peptide. However, the role of RALF1-FER in the vegetative-reproductive growth transition remains unknown. Here, we analyze the mechanism through which FER affects the flowering time in *Arabidopsis.*

**Results:**

We found that the *FER* mRNA levels exhibit an oscillating pattern with a diurnal rhythm and that the clock oscillator *CIRCADIAN CLOCK-ASSOCIATED1* (*CCA1*) up-regulates the expression of *FER* by associating with its chromatin. In addition, *FER* expression is regulated by clock genes, and FER also modulates the expression patterns of clock genes. Consistent with its gene expression pattern, FER positively regulates flowering by modulating the transcript accumulation and mRNA alternative splicing of certain flowering-related genes, including FLOWERING LOCUS C (FLC) and its homolog MADS AFFECTING FLOWERING (MAF)*.* However, the RALF1 ligand negatively regulates flowering compared with FER.

**Conclusions:**

We found that FER, which is up-regulated by CCA1, controls the flowering time by regulating the transcript accumulation and mRNA alternative splicing (AS) of some important flowering genes, and these findings link FER to the floral transition.

## Background

The circadian clock is a typical mechanism that is synchronized by both endogenous and external signals to regulate the vegetative-reproductive growth transition. The circadian clock consists of multiple interlocking feedback loops [1–3]. Briefly, two transcription factors, e.g., *CIRCADIAN CLOCK-ASSOCIATED 1* (*CCA1*) and *LATE ELONGATED HYPOCOTYL* (*LHY*), are key components of the circadian clock and suppress the expression of *PSEUDO-RESPONSE REGULATOR 7* (*PRR7*) and *PRR9.* In turn, *PRR7* and *PRR9* repress the mRNA accumulation of *CCA1* and *LHY*, resulting in the formation of the morning loop [4, 5]. In the central loop, *CCA1* and *LHY* inhibit expression of the transcriptional repressor *TIMING OF CAB EXPRESSION 1* (*TOC1*) [6], whereas the expression of *TOC1* down-regulates the expression levels of *CCA1* and *LHY* [7, 8]*.* In the evening loop, *TOC1* represses the expression of *GIGANTEA* (*GI*) [8], and the evening complex (EC), which includes EARLY FLOWERING 3 (ELF3), ELF4 and LUX ARRHYTHMO (LUX), suppresses the expression of *TOC1*, *GI*, and *PRR9* [9, 10]. These three interlocking feedback loops are the fundamental components of the circadian clock.

Under long-day (LD) conditions, the circadian clock regulates the flowering time via the *GI-CONSTANS* (*CO*)*-FLOWERING LOCUS T* (*FT*) pathway, which is called the photoperiodic pathway [11–15]. CO is a zinc finger transcription factor that promotes flowering by directly activating *FT* expression [16, 17]. FT is the key regulator of the floral pathway, and diverse inputs, including *FLC*, are integrated by regulating the expression of *FT* [18]. FLC, a MADS-box transcription factor, has five homologs, MAF1 to MAF5, in *Arabidopsis*. *FLC* acts as a key repressor of flowering via the vernalization and autonomous pathways by inhibiting the expression of *FT* and *SUPPRESSOR OF CONSTANS 1* (*SOC1*). The mRNA expression of *FLC* is regulated by several genes, including *FCA*, *FY*, *FLK*, *FLD*, *VRN1*, *VRN2* and *VIN3*, in both the autonomous and vernalization pathways [19]. In addition, increasing lines of evidence have shown that *FLC* precursor pre-mRNA processing plays an important role in controlling the flowering time. For example, the SC35 and SCL proteins regulate *FLC* splicing to control the flowering of *Arabidopsis* [20]. The RNA-binding proteins RZ-1B and RZ-1C have been implicated in the regulation of FLC splicing through their interaction with serine/arginine-rich (SR) proteins [21]. AtU2AF65b functions in abscisic acid-mediated flowering by regulating the alternative splicing of FLC [22]. All these data indicate that the regulation of *FLC* pre-mRNA and mRNA expression is essential for flowering.

Alternative splicing (AS), which refers to the production of multiple mRNA isoforms from a single gene, regulates gene expression and increases protein diversity in eukaryotes. AS is mediated by the spliceosome, which consists of five small nuclear ribonucleoprotein particles (snRNPs) and more than 180 types of proteins [23]. Some studies have indicated that AS regulates the timing of floral transition through the integration of external environmental signals, including the ambient temperature and environmental stress. For example, cold treatment affects the abundance of two different *MAF2* splice variants, *MAF2 var1* and *MAF2 var2*, which have different functions in the modulation of the flowering time [24]. In addition, FLOWERING LOCUS M (FLM) has two main splice variants, FLM-β and FLM-δ, which compete for interaction with the floral repressor SHORT VEGETATIVE PHASE (SVP). SVP interacts with the protein splice variant FLM-β to repress flowering at low temperatures. In contrast, the DNA-binding ability of the SVP-FLM-δ complex is reduced at high temperatures to accelerate flowering [25]. Protein arginine methyltransferase 5 (PRMT5/SKB1), known as shk1 kinase-binding protein, is essential for pre-mRNA splicing. This protein dissociates from the FLC promoter, and salt stress induces increases in FLC expression, which results in late flowering [26]. External environmental signals modulate AS-induced flowering. However, the receptors that can link external environmental signals with regulation of the AS of flowering genes remains largely unclear.

The receptor-like kinase FERONIA (FER), which serves as a node for the crosstalk between plant growth and environmental cues, is a versatile regulator of plant growth and survival [27–29]. Glycosylphosphatidylinositol-anchored proteins (GPI-APs), namely, LRE (LORELEI) and LLG1 (LRE-like GPI-APs), which act as chaperones/co-receptors of FER, work together with FER after LLG1/LER-FER perceives different RALF peptides [30–32]. The loss-of-function mutation of *fer-4* causes defects in cell elongation, leading to retarded vegetative development and shorter root hairs [33, 34], which indicates that FER promotes cell growth in some vegetative tissues. In addition, FER is involved in stress responses, such as responses to temperature, salt, and pathogens [27, 29, 35–37]. Therefore, FER controls cell growth and the stress response by integrating different environmental cues and endogenous factors in *Arabidopsis*. In this study, we found that FER, which is up-regulated by CCA1, controls the flowering time by regulating the transcript accumulation and mRNA AS of some important flowering genes and is thus linked to the floral transition.

## Results

### FER transcripts oscillate at a diurnal rhythm controlled by CCA1

**Fig. 1:**
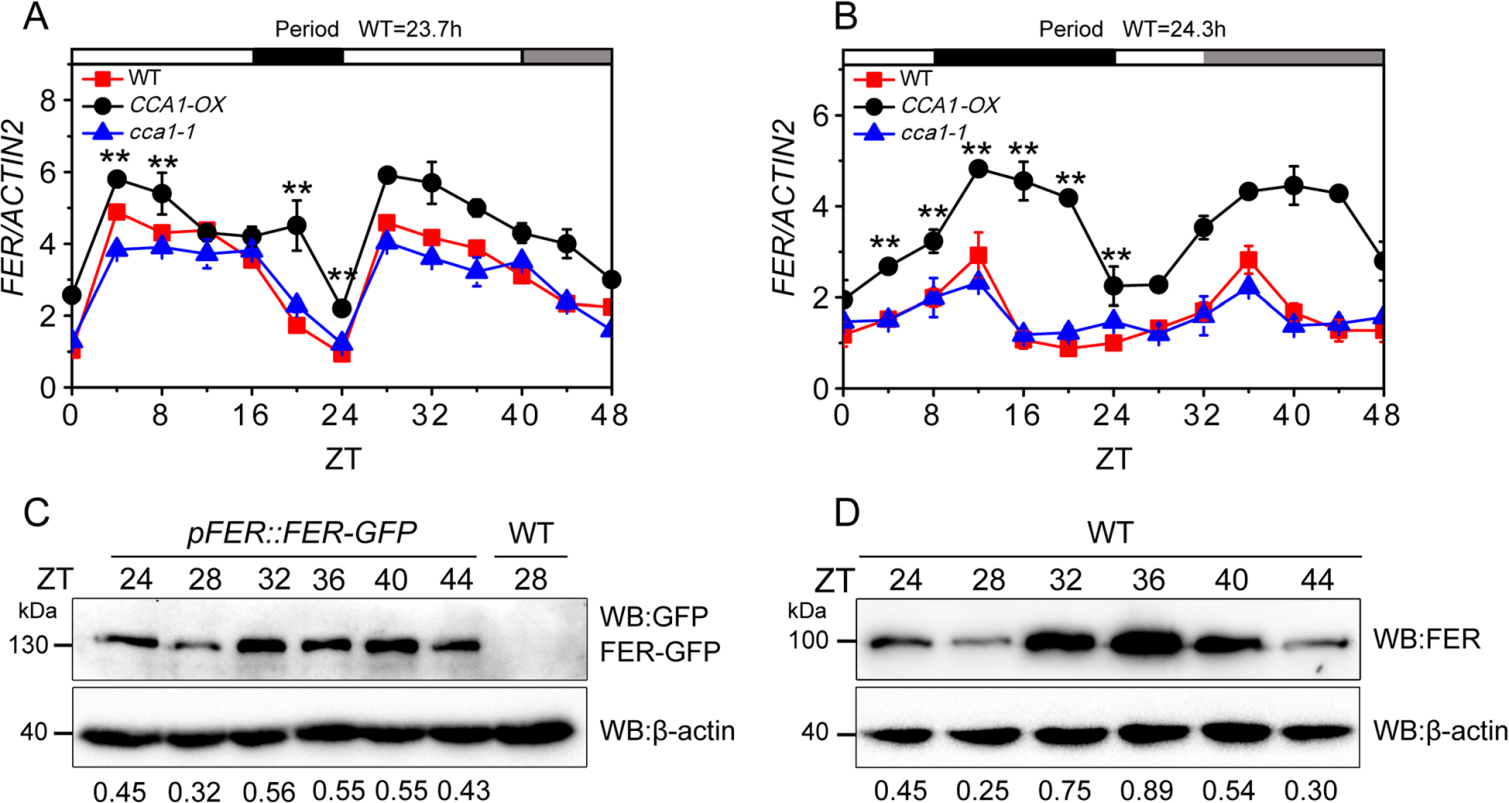
*FER* expression oscillates at a diurnal rhythm. **a** *FER* expression in *CCA1-OX*, *cca1–1* and WT plants under LD condition. Seedlings sampled at 4-h intervals were analyzed by qPCR. Day, night, and subjective night are denoted by white, black, and gray bars. ZT 0 represents the light-on time of the day, during which the sample was collected. *ACTIN2* was used as an internal control to calculate the relative mRNA levels; the experiments were repeated three times, and the error bars represent the SD of three technical replicates. The period was analyzed using BioDare2. Asterisks indicate a significant difference between *CCA1-OX* and WT (**P < 0.01, one-way ANOVA with Tukey’s test). **b** *FER* expression in *CCA1-OX*, *cca1–1* and WT plants under SD condition. The experiments were repeated three times, and the error bars represent the SD of three technical replicates. The period was analyzed using BioDare2. Asterisks indicate a significant difference between *CCA1-OX* and WT (**P < 0.01, one-way ANOVA with Tukey’s test). **c** Immunoblot analysis. The samples were collected at 4-h intervals. All proteins were extracted from *pFER::FER-GFP* and WT seedlings and then analyzed using anti-GFP antibodies to detect the FER-GFP protein. The FER-GFP/β-actin ratio is displayed below the gel, and β-actin was used as the loading control. The experiments were independently repeated three times with similar results. **d** Immunoblot analysis. Protein was extracted from WT seedlings and then analyzed using anti-FER antibodies to detect the FER protein. β-actin was used as the loading control, and the FER/β-actin ratio is displayed below the gel. At least three biological replicates were performed, and similar results were obtained.

We hypothesized that *FER* might be controlled by a diurnal rhythm because the transcript levels of *FER* gene depended on the sampling time. Thus, we analyzed the expression of *FER* using the web-based tool “Diurnal” (http://diurnal.mocklerlab.org/diurnal_data_finders/new) [38]. The results showed that *FER* exhibits a diurnal rhythm under LD, short-day (SD) and continuous-light (LL) conditions (Fig. S1A-D). According to the qPCR analysis, the expression of *FER* fluctuated rhythmically after transfer from 16-h light/8-h dark (LD) conditions to LL conditions and from 8-h light/16-h dark (SD) conditions to LL conditions (Fig. 1a-b). The *FER* oscillation lasted approximately 24 h. After transfer from LD to LL conditions, the transcript level of *FER* first increased at dawn, peaked in the morning, and reached its maximum and minimum during Zeitgeber time 32 (ZT 32) and ZT 28, respectively (Fig. 1a). In contrast, after transfer from SD to LL conditions, the expression of *FER* increased during daytime and peaked at ZT 40 (Fig. 1b). To readily assess the total FER protein (including the phosphorylated and dephosphorylated forms) levels in one band, we shortened the SDS-PAGE running time to prevent separation of the phosphorylated and dephosphorylated forms of FER [27]. According to a GFP expression analysis, the lowest and highest accumulation of the FER protein in the *pFER:FER-GFP* tagged plants was detected at ZT 28 and ZT 32, respectively (Fig. 1c). A similar result was obtained with the WT plants using the FER antibody (anti-FER) (Fig. 1d). The protein expression of FER was consistent with the mRNA oscillation pattern under LD conditions, and an approximately 4-h delay was observed between FER protein and mRNA expression because protein translation generally occurs after mRNA transcription. To determine whether the expression of *FER* is controlled by *CCA1*, we measured the *FER* mRNA levels in WT, *CCA1-*overexpressing (*CCA1-OX*) and *cca1–1* mutant plants under LD and SD conditions. The *FER* mRNA levels oscillated in both the *CCA1-*overexpressing line (*CCA1-OX*) and the *cca1–1* mutant. The expression level in the *CCA1-OX* plants was higher than that in the WT plants under both LD and SD conditions (Fig. 1a and b), which suggested that the expression of *FER* is affected by *CCA1*.

### CCA1 regulates FER expression by directly binding to its chromatin

**Fig. 2:**
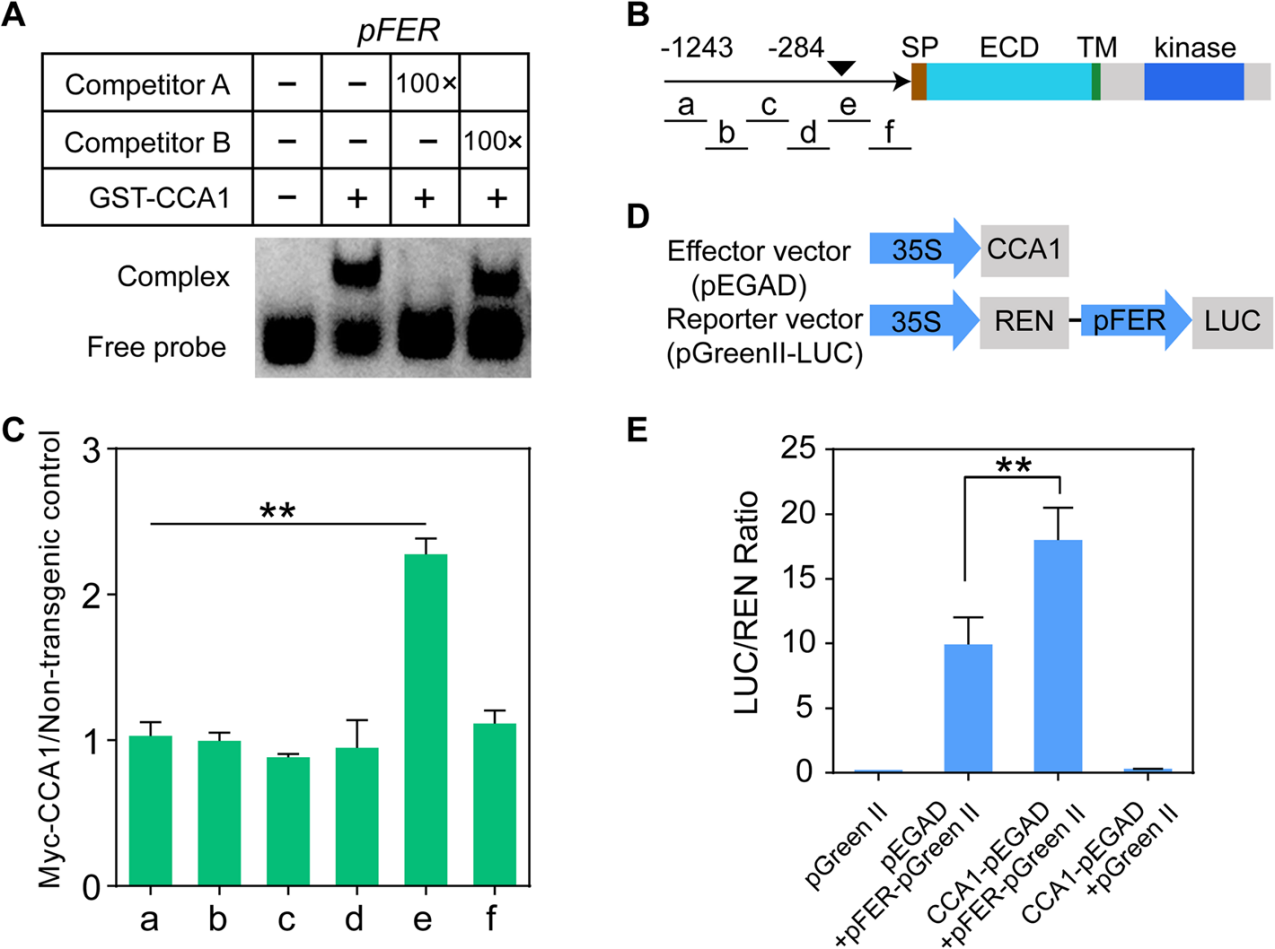
CCA1 interacts with the chromatin regions of FER and promotes its expression. **a** EMSA was performed to confirm the binding of CCA1 to the EE motif of the FER promoter in vitro. Competitor A was the CCA1 binding motif without FITC labeling; Competitor B was the mutant EE fragment without FITC labeling. pFER: The FER fragment contained putative CCA1 binding sites. Sequences of individual DNA probes are listed in Additional file 13: Table 5. The experiments were independently repeated four times with similar results. **b** Diagram depicting the promoter (arrow), signal peptide (SP), extracellular domain (ECD), transmembrane domain (TM) and kinase domain (Kinase). The black triangles indicate the positions of the EE element (AAATATCT), and the black solid lines depict the DNA regions that were amplified by ChIP-qPCR. **c** ChIP-qPCR. ChIP assays were performed using an anti-Myc antibody. DNA regions that were amplified by qPCR are indicated by black solid lines in B. Plants were grown under LD conditions and harvested at ZT 0. Values are relative to a non-transgenic control, and three independent experiments were performed with similar results, error bars represent the SD of three biological replicates (**P < 0.01, Student’s t-test). **d** Reporter and effector constructs used in the transient expression assays. REN luciferase was used as an internal control to normalize the values in individual assays. **e** Relative reporter activity (LUC/REN) in *Arabidopsis* protoplasts. Error bars represent the SD of three biological replicates, and asterisks indicate a significant difference (**P < 0.01, Student’s t-test).

The mRNA expression levels of *FER* were significantly increased in the *CCA1-OX* plants under both LD and SD conditions (Fig. 1a and b). Because CCA1 is a transcription factor, we speculated that CCA1 might modulate *FER* expression by binding to the *FER* chromatin in *Arabidopsis*. Interestingly, the chromatin of *FER* contained an EE (AAATATCT) element (Additional file 2: Figure S2A and B), which is a candidate binding site for CCA1 under LD conditions [39, 40]. In addition, according to the ChIP-seq data, CCA1 binds to *FER* chromatin [39, 40]. To confirm that CCA1 binds to *FER* chromatin, we purified the GST-CCA1 protein and performed an electrophoretic mobility shift assay (EMSA). As shown in Fig. 2a, CCA1 associates with *pFER*, which contains an EE element. We then performed a competitive EMSA with unlabeled WT and mutant (AAATATCT mutated to GGGGGGGG) probes and found that the WT probe, but not the mutant probe, competed with the labeled probe, which indicated that CCA1 specifically binds to *FER* chromatin*.* We subsequently performed chromatin immunoprecipitation (ChIP) assays using both the WT (Col-0) and *35S::CCA1-Myc* line with the anti-Myc antibody. The fragments of *FER* chromatin containing the EE element were enriched in the *35S::CCA1-Myc* but not the WT plants under the LD conditions, which suggested that CCA1 binds to *FER* chromatin in vivo (Fig. 2b and c). In addition, we performed dual-luciferase (LUC) assays to determine whether CCA1 regulates the expression of *FER* by directly interacting with *FER* chromatin. In this assay, the *CCA1* overexpression construct (*CCA1-pEGAD*) was used as the effector, and the *pFER-LUC* reporter (*pFER-pGreen II*) was the readout (Fig. 2d). As shown in Fig. 2e, the LUC activity in the *CCA1-pEGAD* group was higher than that in the *pEGAD* control group, which indicated that *CCA1* coexpression increases the expression of *FER* in *Arabidopsis*. Thus, CCA1 binds directly to *FER* chromatin and up-regulates *FER* expression in *Arabidopsis*.

### FER mutation alters expression patterns of clock genes

**Fig. 3:**
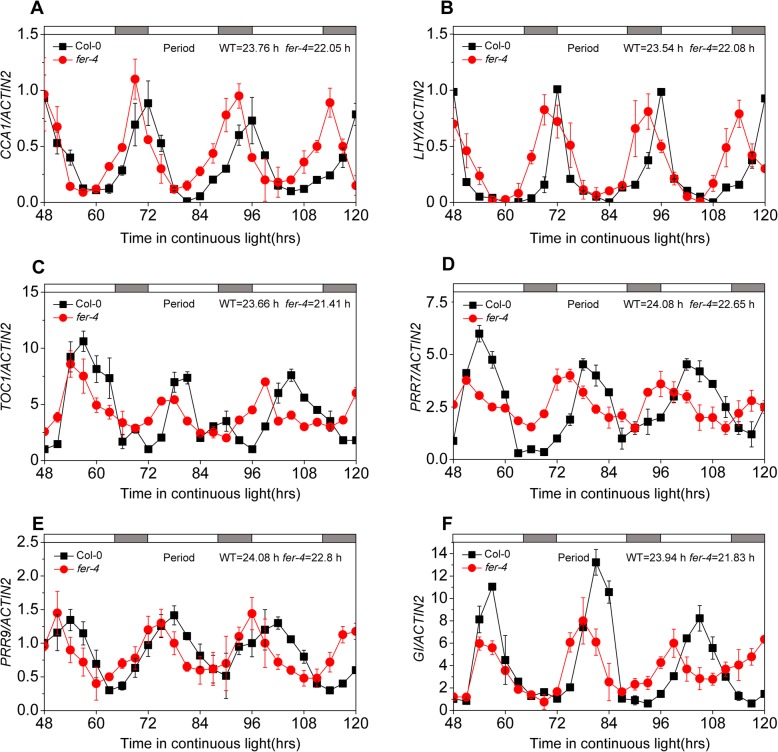
*FER* mutation affects the expression patterns of the clock genes. The expression levels of *CCA1* (**a**), *LHY* (**b**), *TOC1* (**c**), *PRR7* (**d**), *PRR9* (**e**) and *GI* (**f**) transcripts in WT and *fer-4* mutant plants under LL conditions are shown. Seven-day-old seedlings were grown under LD conditions, transferred to LL at ZT 0, sampled at 3-h intervals and analyzed using qPCR. The period was analyzed using BioDare2. All experiments were performed at least three times with similar results, and the error bars represent the SD of three technical replicates

Because the expression of the *FER* gene exhibits a diurnal rhythm in plants, we tested whether the expression of *FER* is a simple output of the clock mechanism or whether *FER*, which is a receptor kinase, also serves as a regulator of the clock mechanism that integrates extracellular signals. The expression levels of six clock genes, namely, *CCA1*, *LHY*, *TOC1*, *PRR7*, *PRR9* and *GI*, in the WT and *fer-4* mutant were measured every 3 h, and these genes were then analyzed using the BioDare2 website [41]. The analysis revealed that the periods of these six genes in the *fer-4* mutant plants were shorter than those in the WT plants (Fig. 3a-f). In addition to shortening the period of the clock genes, the *fer* mutation altered the amplitudes of the *CCA1*, *TOC1* and *PRR7* genes under LD conditions (Additional file 3: Figure S3A-D). The amplitude of *CCA1* increased by more than 70% (amplitude: Col-0 = 0.38 and *fer-4* = 0.68) in the *fer-4* mutant (Additional file 3: Figure S3A). However, the *TOC1* and *PRR7* transcription levels decreased in the *fer-4* mutant. These results indicated that *FER* expression is regulated by clock genes and that FER modulates the expression patterns of clock genes.

### FER modulates the flowering time

**Fig. 4:**
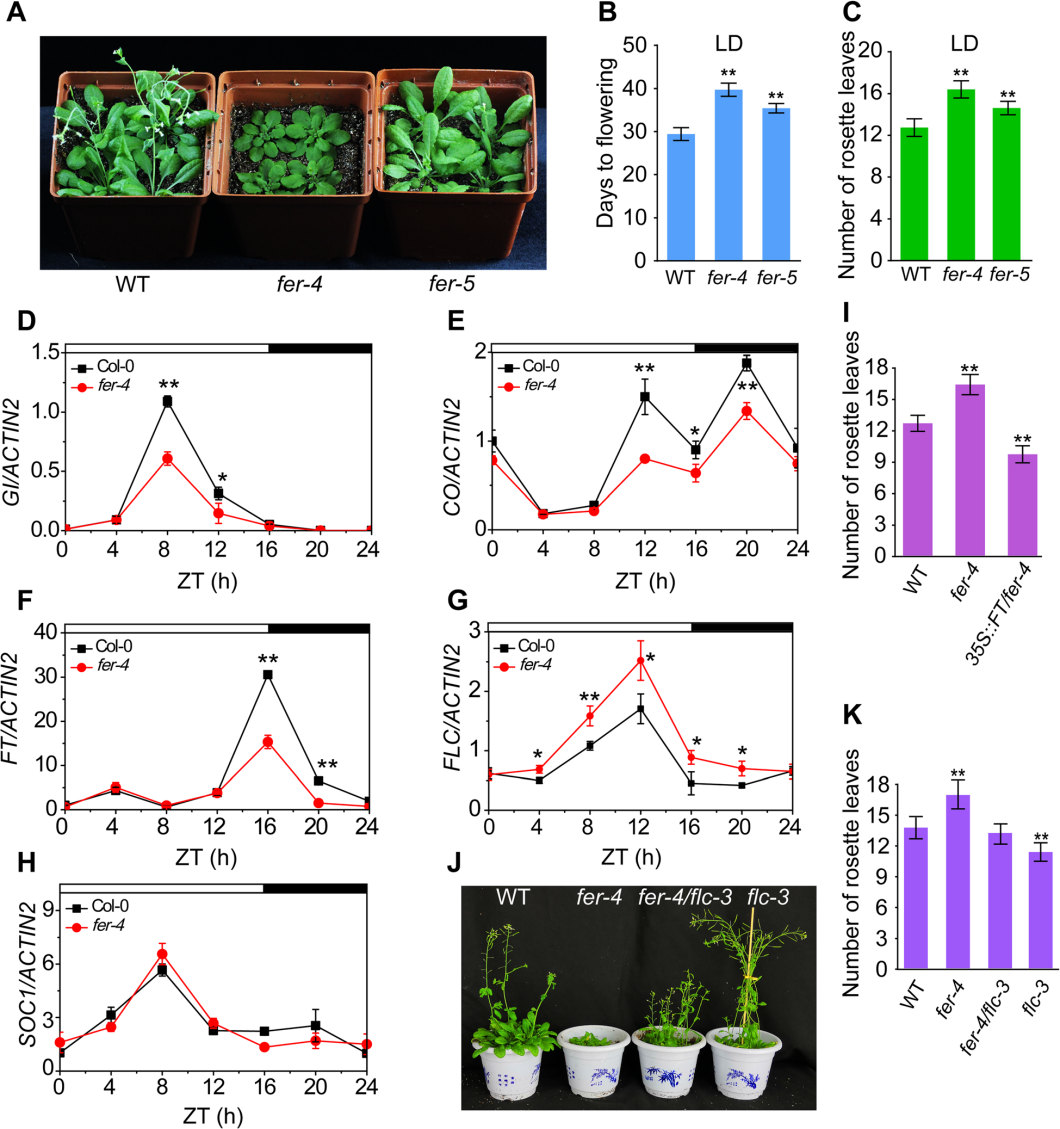
FER regulates the flowering time in Arabidopsis. **a** Photographs of plants of various genotypes grown for 35 days under LD conditions. **b** The flowering times were measured as days to flower under LD conditions. Asterisks indicate a significant difference compared with WT (*n* = 15, **P < 0.01, one-way ANOVA with Tukey’s test). **c** Statistical analysis of leaf numbers of *fer-4* and *fer-5* plants compared with WT plants. Values are the mean ± SD of at least 15 plants (**P < 0.01, one-way ANOVA with Tukey’s test). **d**-**h** Expression levels of *GI* (D), *CO* (E), *FT* (F), *FLC* (G), and *SOC1* (H) transcripts in WT and *fer-4* mutant plants are shown. Fourteen-day-old seedlings were grown under LD conditions and sampled and analyzed by qPCR. Day and night are denoted by white and black bars, respectively. All experiments were performed at least three times with similar results. Values are the mean ± SD (*P < 0.05, **P < 0.01, Student’s t-test). **i** Statistical analysis of the rosette leaf numbers at bolting in WT, *fer-4* and *35S:FT/fer-4* plants grown under LD conditions. The experiments were independently repeated three times with similar results (*n* > 20, **P < 0.01, one-way ANOVA with Tukey’s test). **j** Phenotypes of WT, *fer-4*, *fer-4/flc-3* and *flc-3* grown under LD conditions. **k** Statistical analysis of leaf numbers of different genotypes compared with WT plants (*n* > 20). Error bars represent SD. The experiments were performed at least three times. Asterisks indicate a significant difference compared with WT (**P < 0.01, one-way ANOVA with Tukey’s test)

CCA1 overexpression causes late flowering though the repression of *GI* [42], as high expression of *CCA1* was observed in the *fer-4* mutant (Additional file 3: Figure S3A). We speculated that FER likely plays a role in the control of the flowering time. The flowering times of the *fer-4* mutant and WT plants were examined. Under LD conditions, the *fer-4* mutant showed a significant delay in the flowering time, as measured by either the days to flowering or the numbers of leaves at flowering (Fig. 4a-c). This phenotype was also observed in *srn* [34, 43], which is another *FER-*null mutant in the ecotype C24 background (Additional file 4: Figure S4A-C). We found that *fer-5*, which is a knock-down mutant of FER (Duan et al., 2010), had a late-flowering phenotype (Fig. 4a-c). To determine the molecular mechanism underlying the delayed flowering phenotype of the *fer-4* mutant, we examined the expression of *GI*-*CO*-*FT*, a key pathway of photoperiodic flowering. In the *fer-4* mutant, the expression of *GI* began to increase at ZT 4 and peaked at ZT 8, which is similar to the findings observed in the WT plants. However, the *GI* peak in the *fer-4* mutant was significantly lower than that in the WT plants (Fig. 4d). Similarly, the oscillation pattern of the *CO* transcript in the WT plants was similar to that in the *fer-4* mutant, but the expression level of *CO* was lower in the mutant than in the WT (Fig. 4e), which led to the lower peak in *FT* expression observed in the *fer-4* mutant (Fig. 4f). To further confirm that FER regulates flowering mainly through the *GI*-*CO*-*FT* pathway, we examined the expression of two other key flowering factors, FLC and SOC1. FLC is a center node of the autonomous and vernalization pathways, and FLC expression was significantly elevated in the *fer-4* mutant compared with the WT plants, which indicated that either the autonomous or vernalization pathway is affected in the *fer-4* mutant (Fig. 4g). In addition, the transcript level of *SOC1* was unaffected in the *fer-4* mutant (Fig. 4h). To confirm the genetic roles of FLC and FT that regulate flowering in the *fer-4* mutant, we compared the flowering phenotypes among the Col-0, *fer-4*, *35S:FT/fer-4*, *flc-3* and *fer-4/flc-3* plants. The flowering time of the *35S:FT/fer-4* plant was earlier than those of the *fer-4* mutant and WT plants (Fig. 4i), which indicated that FT expression could recover the late-flowering phenotype of the *fer-4* mutant. Moreover, the loss of function of *FLC* in the *fer-4* mutant background alleviated the late-flowering phenotype of the *fer-4* mutant (Fig. 4j-k), which indicated that FLC is involved in the FER-mediated acceleration of floral transition. Together, these results indicate that FER regulates flowering in *Arabidopsis* through multiple pathways.

### FER regulates the AS of some flowering-related genes

**Fig. 5:**
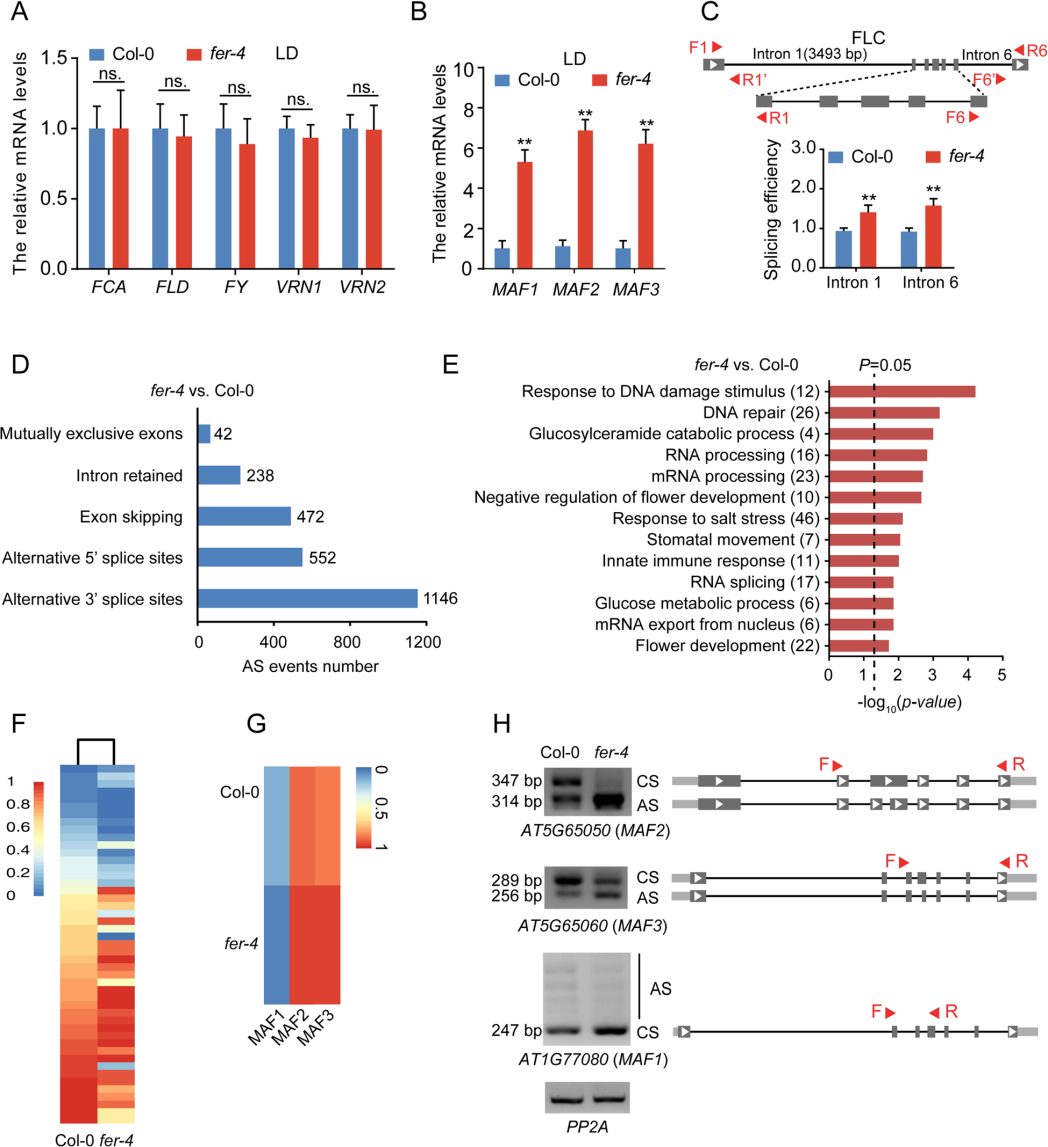
FER is required for pre-mRNA splicing of flowering-related genes in Arabidopsis. **a**-**b** Relative mRNA levels of WT and *fer-4* mutant at ZT 4. LD, long day condition. *ACTIN2* was used as an internal control gene. Error bars indicate the SD of three technical replicates. All experiments were performed at least three times (ns. means no significant; **P < 0.01, Student’s t-test). **c** Genomic structure of FLC. The gray boxes represent the exons; black lines indicate introns; F, forward primer; R, reverse primer; The primer pairs F1/R1’ and F6’/R6 were used to detect the unspliced RNA for FLC introns 1 and 6, respectively. F1/R1 and F6/R6 were used to detect spliced mRNA in wild-type and *fer-4* mutants. *ACTIN2* was used as an internal control. Seven-day-old Col-0 and *fer-4* seedlings were grown under LD conditions and collected at ZT 12. Splicing efficiency (spliced/unspliced) was calculated. The experiments were performed three times (**P < 0.01, Student’s t-test). **d** Numbers of differential splicing events between the *fer-4* mutant and WT detected by RNA sequencing (RNA-Seq) analysis (*n* = 3). **e** Gene Ontology (GO) enrichment of genes with significant splicing changes between *fer-4* and WT (*n* = 3). The black dotted line indicates *P* = 0.05. The numbers indicated the representative genes involved in the pathway. **f** Heat maps of differential pre-mRNA splicing of flowering-related genes between *fer-4* mutant and wild-type based on the exon inclusion levels detected by RNA-Seq analysis. **g** Heat maps of differential pre-mRNA splicing of flowering-related genes (*MAF1*, *MAF2* and *MAF3*) based on the exon inclusion levels from RNA-seq data (*n* = 3). **h** Semi-quantitative PCR (semi-qPCR) validation of differential splicing events between *fer-4* mutant and Col-0. Seven-day-old Col-0 and *fer-4* seedlings were grown under LD conditions and collected at ZT 4. CS indicates constitutive splicing, and AS indicates alternative splicing. Red triangles show the position of primers used by semi-qPCR (Supplemental Table 5). *PP2A* was used as the internal control. The experiments were independently repeated three times with similar results

We subsequently focused on the mechanism through which FLC expression is up-regulated in the *fer-4* mutant compared with the WT plants. There are two possible explanations for the higher expression of FLC observed in the *fer-4* mutant. First, the *FER* mutation might decrease the expression of some regulators of *FLC* to release *FLC* expression, and second, the FER mutation might cause the abnormal splicing of FLC, resulting in an increase in functional FLC mRNA. To explore the silencing mechanism of *FLC*, we examined whether various FLC regulators, including *FLOWERING CONTROL LOCUS A* (*FCA*), *FLOWERING LOCUS D* (*FLD*)*, FY*, *VERNALIZATION 1* (*VRN1)* and *VRN2*, were affected in the *fer-4* mutant. The results showed that the mRNA expression levels of these genes showed only a slight little difference in the *fer-4* mutant compared with the WT plants, which indicated that the increased FLC expression observed in the *fer-4* mutant was not caused by these genes (Fig. 5a). We also assessed the mRNA levels of three homologs of FLC, namely, *MAF1*, *MAF2* and *MAF3*. The expression levels of these genes were significantly increased in the *fer-4* mutant compared with the WT plants (Fig. 5b), which suggested that *MAF* homologs likely act as targets of FER. We then measured the ratio of spliced to unspliced FLC transcripts to analyze the splicing efficiency of FLC introns 1 and 6 [44]. The splicing efficiency of both introns 1 and 6 was increased in the *fer-4* mutant compared with the WT plants (Fig. 5c and Additional file 5: Figure S5). We predicted that the increased splicing efficiency of *FLC* introns 1 and 6 would result in a decreased level of unspliced RNA but resulted in an increased level of spliced RNA. Thus, the increased level of spliced FLC RNA increases the expression of *FLC* in the *fer-4* mutant compared with the WT plants.

We performed an RNA-Seq analysis to identify additional potential target flower-related genes of FER-mediated splicing. We generated more than 40 million reads, and 89.8% of the generated reads could be properly aligned to the TAIR10 reference genome (Additional file 6: Figure S6A). By plotting the coverage of reads along each transcript unit, we found a uniform distribution with no obvious 3′/5′ bias (Additional file 6: Figure S6B), and a comparison of the mapped reads to the gene model (TAIR10) revealed that approximately 99% of the reads mapped to exons (Additional file 6: Figure S6C), which indicated the high quality of the cDNA libraries. The RNA-Seq analysis revealed that 1753 and 1634 genes displayed higher (at least 2-fold with *P* < 0.05) and lower transcript levels, respectively, in the *fer-4* compared with the Col-0 plants (Additional file 6: Figure S6D and Additional file 9: Table S1). Based on the RNA-Seq data, 2450 genes were identified as having splicing defects in the *fer-4* mutant compared with the WT control (*P* < 0.05, inclusion level difference > 0.05 or < − 0.05), and these could be sorted into five categories: 1146 alternative 3′ splice sites (A3’SS), 552 alternative 5′ splice sites (A5’SS), 472 exon skipping (ES), 238 intron retained (IR) and 42 mutually exclusive exons (MXE) (Fig. 5d and Additional file 10: Table S2). A Gene Ontology term enrichment analysis revealed that different splicing genes are involved in RNA processing, response to salt stress and flower development regulation (Fig. 5e and Additional file 11: Table S3). We identified approximately 28 flower-related genes that might be involved in FER-mediated splicing, and these included MAF1, MAF2, FY and EMBRYONIC FLOWER 2 (EMF2) (Additional file 12: Table S4). To better visualize the differential splicing between *fer-4* and WT in the flowering-related pathway, we used heat maps of the genes showing splicing changes (Fig. 5f), which indicated that the splicing patterns were significantly altered in the *fer-4* mutant. Through semi-qPCR analysis, we also confirmed that some flowering-related genes (*MAF1*, *MAF2*, and *MAF3*) exhibit splicing changes (Fig. 5g and h and Additional file 13: Table S5). In summary, these results support a function of FER in regulating flowering in *Arabidopsis* by mediating AS.

### RALF1 peptide affects the flowering time under LD conditions

**Fig. 6:**
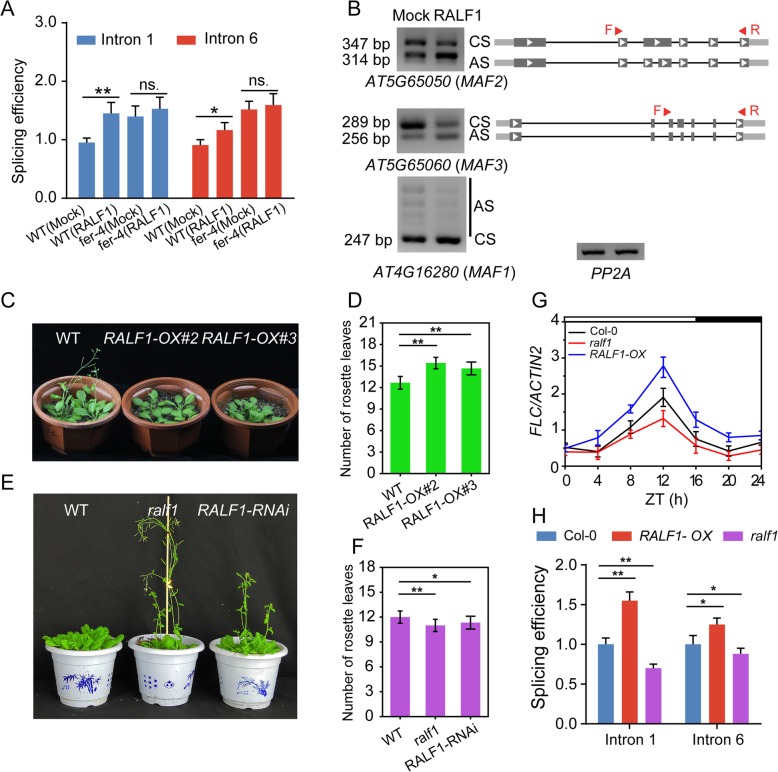
RALF1 regulates the flowering time. **a** qPCR analysis of the splicing efficiency of FLC introns 1 and 6 in the seedlings of wild-type and *fer-4* mutants with or without RALF1 treatment. The samples were collected at ZT 4. The bar indicates the mean ± SD, and the experiments were independently repeated three times (ns. means no significant; *P < 0.05, **P < 0.01, Student’s t-test). **b** Different splicing variants of *MAFs* in Col-0 with or without RALF1 treatment as determined by semi-qPCR analysis. CS indicates constitutive splicing, and AS indicates alternative splicing. mRNA was isolated from 7-d-old seedlings and sample were collected at ZT 4. The results were repeated three times with similar result. **c** The flowering genotypes of wild-type and *RALF1-OX* plants under LD conditions. **d** Number of rosette leaves in WT (*n* = 15) and *RALF1-OX* (*n* = 15) under LD conditions. Data are presented as the mean ± SD, and the experiments were independently repeated three times with similar results (**P < 0.01; one-way ANOVA with Tukey’s test). **e** The flowering genotypes of wild-type, *ralf1* and *RALF1-RNAi* grown under LD conditions. **f** Flowering time was measured by the number of rosette leaves at bolting. Error bars indicate the SD (*n* = 15). The experiments were independently repeated three times with similar results. Statistically significant differences are denoted with asterisks (*P < 0.05, **P < 0.01; one-way ANOVA with Tukey’s test). **g** Expression level of *FLC* detected by qPCR in 7-d-old Col-0, *RALF1-OX* and *ralf1* mutant seedlings. Error bars indicate the SD of three technical replicates. Day and night are denoted by white and black bars, respectively. The experiments were performed at least four times. **h** Splicing efficiency of FLC intron 1 and intron 6 in the seedling of Col-0, *RALF1-OX* and *ralf1* mutant. Seedlings were grown under LD conditions for 7 days and collect and ZT 12. The primer pairs F1/R1’ and F6’/R6 were used to detect the unspliced RNA for FLC introns 1 and 6, respectively. Primer pairs F1/R1 and F6/R6 were used to detect the spliced mRNA. The experiments were performed three times, the bar indicates the mean ± SD (*P < 0.05, **P < 0.01,one-way ANOVA with Tukey’s test)

FER functions as a receptor for RALF1 and RALF23 [31, 32], and in this study, FER was shown to be involved in the control of the AS of some flowering-related genes. We tested whether RALF1 or RALF23 might serve as a peptide hormone that alters the splicing pattern of flowering genes by activating FER. First, we analyzed the expression of RALF1 and RALF23 in ePlant and found that both were expressed in the shoot apex (Additional file 7: Figure S7A-B). A qPCR analysis further confirmed that RALF1 is expressed in the shoot (Additional file 7: Figure S7C). We subsequently used RALF1 as an example to analyze the role of RALF peptides in the control of flowering. The WT and *fer-4* mutant plants were treated with the RALF1 peptide, and the splicing efficiencies of FLC introns 1 and 6 were detected. The efficiencies of the FLC introns 1 and 6 significantly increased after RALF1 treatment in Col-0 plants, whereas the alterations in the splicing efficiencies of FLC introns 1 and 6 caused by RALF1 treatment were significantly attenuated in the *fer-4* mutant (Fig. 6a). To investigate whether the pre-mRNA splicing of MAFs is regulated by RALF1, we analyzed the splicing changes in MAFs with or without RALF1 treatment. The results showed that the splicing pattern of MAF1–3 was altered in the RALF1-treated plants, similar to the results obtained in the *FER* mutant plants, which indicated that FER and RALF1 might have opposite roles in regulating the AS of some flowering genes (Fig. 6b). We subsequently assessed the role of RALF1 in regulating the flowering time in *Arabidopsis*. First, we obtained two *RALF1-*overexpressing lines (*RALF1-OX#2* and *RALF1-OX#3*) and identified two *ralf1* mutants (*ralf1*-knockout and *RALF1-RNAi*-knockdown lines) (Additional file 8: Figure S8A-D). Under LD conditions, the two *RALF1-OX* lines showed a late-flowering phenotype compared with the WT line (Fig. 6c-d). In contrast, the *ralf1* mutant and *RALF1-RNAi* line displayed an early-flowering phenotype (Fig. 6e-f). The mRNA levels of *FLC* increased in *RALF1-OX* and decreased in the *ralf1* mutant, which was consistent with the flowering phenotype (Fig. 6g). In addition, the splicing efficiencies of introns 1 and 6 were increased in the *RALF1-OX* plants and decreased in *the ralf1* mutant compared with WT. In summary, these results indicate that RALF1 also regulates the splicing of flowering genes and exerts an opposite effect on the flowering time compared with FER.

## Discussion

**Fig. 7:**
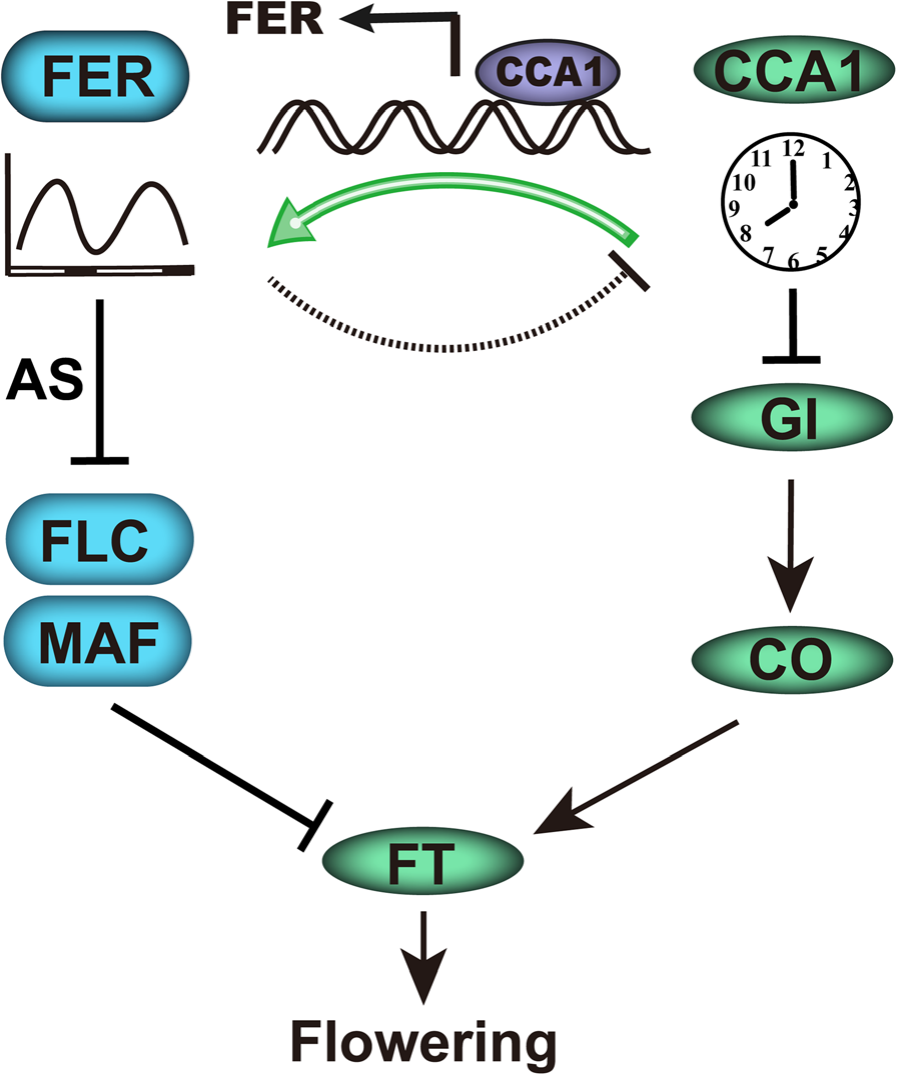
Proposed working model of FER function in *Arabidopsis* flowering. *FER* fluctuated at a diurnal rhythm along with FER, and the clock oscillator CCA1 appears to bind the FER chromatin and promote its expression. In turn, FER will down-regulate CCA1 expression and regulate flowering through the GI-CO pathway. Thus, FER-CCA1 appears to form an interlocking feedback loop during flowering time control. In addition, FER is a negative regulator of FLC and MAF by regulating their splicing and transcript accumulation. Thus, FER functions in flowering transition by splicing pre-mRNA of genes and regulating expression of flowering-related genes. Arrows denote activation, and bars indicate repression. Details are provided in the text

Several environmental signals entrain the circadian oscillator. In this study, we found that FER acts not only as a simple output of the clock but also as a regulator of clock genes. FER mutation affects the period length and amplitude of circadian outputs (Fig. 3a-f and Additional file 3: Figure S3A-D) and thus represses the downstream *GI* transcript and further delays the flowering time partly through the CO-FT pathway (Fig. 7). FER is also involved in other flowering pathways, primarily by repressing FLC and FLC-related MAFs (Fig. 7).

The circadian clock coordinates responses to multiple environmental challenges that cannot be avoided by a sessile plant. More importantly, the strength of many cell signaling pathways is regulated by the circadian clock, and this process is known as circadian gating [45]. Thus, the circadian clock is important for plant growth and fitness. Here, we showed that *FER* fluctuates along with the diurnal rhythm (Fig. 1a-b). Why does *FER* exhibit a rhythm in plant cells? FER is a versatile regulator of plant growth and stress responses [28, 37] and acts as a critical node for the crosstalk of extracellular or intracellular cues [29]. We propose that the diurnal rhythm of *FER* might function as an important output of circadian gating and confers a fitness advantage. For example, FER mediates the inhibition of the immune response in plants [32], and its mRNA nadir occurs at approximately midnight and dawn, which are times that are mainly characterized by sporulation and spore dissemination [46, 47]. This finding is consistent with the hypothesis that the FER diurnal rhythm might be involved in the defense response. In addition, starch accumulates in daytime and degrades at night, and FER also regulates starch metabolism in leaves [48]. We observed that the expression of *FER* begins to increase at dawn, peaks at ZT 4, and then decreases at dusk (Fig. 1a), which is consistent with the hypothesis that the diurnal rhythm of FER might also be related to starch metabolism. More importantly, the diurnal rhythm of *FER* might endow FER with the ability to balance the vegetative and reproductive growth times in response to changes in environmental signaling.

Some studies have indicated that RALFs play opposite roles in certain tissues and/or environmental responses compared with FER. For example, *RALF22/23-*overexpressing transgenic plants and *fer-4* mutants display similar retarded growth phenotypes and increased sensitivity to salt stress [37]. RALF1 and FER play negative and positive roles in leaf growth, respectively [33, 49]. Here, we found that RALF1 and FER have different roles in flowering and the AS of certain mRNAs. One possible reason for this phenomenon is that FER might recruit distinct downstream factors to fulfill its context-specific roles. In addition, RALF23, as another ligand of FER [32], is also expressed in the shoot apex. Further studies should explore whether RALF23 and FER play the same roles in flowering regulation.

The AS of pre-mRNA is an important regulatory mechanism. A few proteins or signaling pathways reportedly regulate AS in plants. Our study suggests that the RALF1 peptide influences the AS of flowering-related genes through FER and further regulates the flowering time. However, the mechanisms used by the RALF1-FER pathway to regulate mRNA AS remain poorly understood. In addition, the mechanism through which the AS of many mRNAs is regulated by the RALF1-FER axis and the physiological significance of RALF1-FER-regulated mRNA AS remain unknown, and these processes should be examined in further studies.

## Conclusions

Here, we found that FER, which is up-regulated by CCA1, not only outputs the clock but also regulates clock genes. This protein controls the vegetative-reproductive growth transition, likely by regulating the mRNA expression and AS of flowering-related genes, including FLC and its homologs (MAFs)*.*

## Methods

### Plant growth conditions

*Arabidopsis* (Columbia ecotype) was used as the WT in all experiments unless stated otherwise. The *fer-4, flc-3* mutant, *35S::FT*, *Ubi::FER-Flag* (*FER-OX*) and *srn* have been previously described [27, 43, 50]. *CCA1-OX* was provided by Paul P. Dijkwe [51], and the *ralf1* mutant (SALK_036331) [52] was obtained from ABRC and confirmed using specific primers (Additional file 8: Figure S8A-B and Additional file 13: Table S5). For the *Ubi::RALF1-Flag* transgenic plants (*RALF1-OX*), the full-length CDS of RALF1 was sub-cloned into the pCAMBIA 1301 vector under the control of the *Ubi* promoter through homologous recombination of the attB1 and attB2 sites (Gateway recombination cloning technology). The *RALF1-OX* lines were generated using the *Agrobacterium-*mediated floral dip method and identified using specific primers (Additional file 8: Figure S8C-D and Additional file 13: Table S5).

For analysis of clock gene expression, the seeds were planted on 1/2 MS medium supplemented with 1.0% sucrose and 0.8% agar (pH 5.8) at 4 °C for 3 days in the dark. The seeds were then transferred to either LD (16-h light/8-h-dark) or SD (8-h-light/16-h-dark) conditions at a light intensity of 50 to 60 μmol m^− 2^ S^− 1^ and a constant temperature of 22 °C. Seven-day-old seedlings grown under LD/SD condition and then transferred to constant light conditions at ZT 24 for FER rhythm analysis.

### RNA extraction and qPCR analyses

For the mRNA expression analyses, the samples were harvested and powdered in liquid nitrogen. Total RNA was extracted using the TRIzol reagent (Ambion, 15,596–026) and digested using DNase I (TaKaRa) to remove the genomic DNA. cDNA was synthesized from 1000 ng of total RNA by using a cDNA synthesis kit (Fermentas, K1622). qPCR was performed using Mx3000P (Stratagene) with SYBR Premix *Ex* Taq II (TaKaRa). The primers used for the qPCR analysis are listed in Additional file 13: Table S5, and *ACTIN2* was used as an internal reference in the circadian experiments. The cDNAs were amplified following denaturation using 42-cycle programs (95 °C, 15 s; 60 °C, 20 s per cycle).

The expression of the *FER*-related gene subfamily was investigated using the web-based tool “Diurnal”, the gene expression data under SD were according to COL_SD (*Arabidopsis* Col-0 grown under circadian conditions of SD), and the gene expression data under LD were according to long day conditions (*Arabidopsis* Ler strain, grown under the circadian conditions of LD). The gene expression data under LL were according to LL23_LDHH [*Arabidopsis* Col-0 grown under the circadian conditions light (12 h), light (12 h)/hot (24 h), and subjected to light (24 h)]. The period and amplitude of the clock genes were analyzed using BioDare2 (https://biodare2.ed.ac.uk) and the FFT NNLS method [41].

### Semi-qPCR analyses

Seven-day-old Col-0 and *fer-4* seedlings were grown at 22 °C under LD conditions and collected at ZT 4. For the RALF1 peptide treatment experiment, 7-d-old seedlings were treated with 1/2 liquid MS in the presence or absence of 1 μM RALF1 for 3 h, then collected at ZT 4 and powdered in liquid nitrogen for RNA extraction. For the semiquantitative PCR (semi-qPCR) analysis of the AS of the flowering genes (e.g., *MAF1*, *MAF2*, and *MAF3*), *Protein phosphatase 2A* (*PP2A*) was used as a reference gene as described in the RNA splicing experiments [53]. The primers for FLC intron retention 1 and 6 were according to Xiong et al. [22]. Semi-qPCR mixes were prepared using 2× Master Mix (TsingKe, TSE004) according to the manufacturer’s instructions. Reactions were performed with initial incubation at 95 °C for 10 min followed by 26 cycles of 15 s at 95 °C, 30 s at 55 °C and 1 min/kb at 72 °C. The semi-qPCR products were electrophoresed on 1.5% agarose gels and stained with ethidium bromide. The primer sequences used for the semi-qPCR analysis are shown in Additional file 13: Table S5.

### Determination of the flowering time

For the rosette leaf measurement, the plants were grown in a randomized fashion on soil under LD conditions at 22 °C in a greenhouse with light intensity conditions of 80–100 μmol m^− 2^ S^− 1^. More than 40 plants of each genotype were planted for each independent experiment. The flowering time was determined according to the visible flower buds at the center of the rosette and the days from germination to flowering from three biological replicates.

### Western blot analysis

For analysis of the protein levels under LD conditions, the WT and *pFER::FER-GFP* plants were planted on 1/2 MS medium for 7 d. The samples were harvested and powdered in liquid nitrogen. Next, 200 mg of the seedlings was extracted with buffer (50 mM Tris-HCl pH 7.5, 150 mM NaCl, 5.0% glycerin, EDTA-2Na, 1.0% Triton X-100) for 30 min and then separated by 10.0% SDS-PAGE to detect the two forms of FER (phosphorylated and non-phosphorylated) in one band. In contrast to our previous study, we did not add glycerol to the SDS-PAGE gel [27], and we shortened the running time to easily survey the total FER level in a single band. The gel was blotted onto an NC filter membrane using tank transfer. The blots were blocked with 5.0% defatted milk for 1 h at 4 °C with agitation, incubated with anti-GFP antibody (CMC, 1:5000) and FER antibody (1:3000) for 4–5 h at RT with agitation, and then washed 3 times for 7 min each with TBS-T at RT with agitation. The blots were incubated with a secondary antibody diluted 1:10000 in milk for 1 h at RT with agitation for enhanced chemiluminescence detection (Thermo Scientific, 34,075). β-actin was used as the loading control.

### Gene cloning and dual-LUC analysis

Genomic DNA was extracted from *Arabidopsis* leaves using the DNeasy Plant Mini Kit (Qiagen, 69,104), the promoter of *FER* (*pFER*) was cloned from the genomic DNA, and the coding region of the *CCA1* gene was cloned from the cDNA. The gene-specific primers used for the PCR analysis are listed in Additional file 13: Table S5. The amplification product of *pFER* was cloned into pGreenII-LUC at the *Bam*HI and *Nco*I sites to add the firefly LUC reporter gene, and *CCA1* was cloned into pEGAD to generate the 35S::CCA1-pEGAD effector [54]. In addition, the pGreenII-LUC vector carrying Renilla (REN) LUC under the control of the 35S promoter served as an internal control. The binding ability of CCA1 to the *FER* promoter was assessed according to the ratio of LUC to REN. The reporter and effector vectors were co-transformed into *Arabidopsis* protoplasts using the PEG method as previously described [43]. After incubation at 22 °C for 24 h, the transformed protoplasts were assayed for LUC and REN using dual LUC assay kits (Promega, E1910). The readouts of LUC and REN were assessed using Fluoroskan Ascent FL (Thermo Scientific, China) according to the manufacturer’s instructions.

### EMSA

For the EMSA, recombinant proteins of GST-CCA1 were expressed in *Escherichia coli* BL21 and subsequently purified using GST beads. The DNA-protein binding reaction was performed by incubating 20 fmol of FITC-labelled probe with 5 μg purified GST-CCA1 protein. For the competition experiments, 5 μg purified GST-CCA1 protein and 100× unlabeled competitor A or 100× nonspecific competitor B were incubated in binding buffer (100 mM Tris-HCl pH = 8.0, 5 mM DTT, 2.5 mM EDTA, 0.25% Triton X-100 and 25% glycerin) for 15 min, followed by the addition of 20 fmol of FITC-labeled probe for 20 min. Next, the binding reaction mixture was loaded onto the 4% PAGE gel (without SDS), which was resolved in 0.5× TBE buffer for 40 min and then exposed to a fluorescence imager plate.

### ChIP assays

The ChIP assay was performed as previously described [55]. Briefly, 3-week-old Col-0 and *35S::CCA1-Myc* grown on soil under LD conditions were collected at ZT 0 and treated with 37 mL extraction buffer 1 (0.4 M sucrose; 10 mM Tris-HCl, pH 8.0; 5 mM β-ME; 0.1 mM PMSF) containing 1% formaldehyde under a vacuum for 15 min. A final concentration of 0.125 M glycine was added to quench the crosslinking, and the vacuum was applied for an additional 5 min. The plants were rinsed twice with water, ground to a powder with liquid nitrogen, and homogenized in extraction buffer 1 [0.4 M sucrose; 10 mM Tris-HCl, pH 8.0; 5 mM β-ME; 0.1 mM PMSF; complete protease inhibitor cocktail tablets (Roche)]. The filtered solution was centrifuged at 2100 g for 20 min at 4 °C, and the pellet was resuspended in 1.5 mL of extraction buffer 2 [0.25 M sucrose; 10 mM Tris-HCl pH 8.0; 10 mM MgCl_2_, 1% Triton X-100; 0.1 mM PMSF; 5 mM β-ME; complete protease inhibitor cocktail tablets (Roche)]. The samples were centrifuged at 12000 g for 10 min at 4 °C and lysed in Nuclei Lysis Buffer [50 mM Tris-HCl pH 8.0, 10 mM EDTA, 1% SDS, 0.1 mM PMSF, and protease inhibitor cocktail tablets (Roche)]. The chromatin solution was sonicated to shear the DNA to 200–600 bp, the sonicated chromatin suspension was centrifuged at 12000 g for 5 min and the supernatant was diluted 10-fold in ChIP Dilution Buffer [16.7 mM Tris-HCl pH 8.0, 167 mM NaCl, 1.1% Triton X-100, 1.2 mM EDTA, 0.1 mM PMSF, complete protease inhibitor cocktail tablets (Roche)]. The anti-Myc antibody was pre-bound to protein A/G magnetic beads, mixed with the chromatin solution and incubated overnight at 4 °C. The beads were then washed with Low Salt Buffer (50 mM Tris-HCl pH 8.0, 150 mM NaCl, 0.2% SDS, 0.5% Triton X-100, 2 mM EDTA), High Salt Buffer (20 mM Tris-HCl pH 8.0, 500 mM NaCl, 0.2% SDS, 0.5% Triton X-100, 2 mM EDTA), LiCl Washing Buffer (20 mM Tris-HCl pH 8.0, 0.25 M LiCl, 1% NP 40, 1% sodium deoxycholate, 1 mM EDTA) and TE Washing Buffer (10 mM Tris-HCl pH 8.0, 1 mM EDTA). The chromatin fragments were eluted with Elution Buffer (50 mM Tris-HCl pH 8.0, 10 mM EDTA, 1% SDS) and incubated at 65 °C for 12 h. The eluate was treated with proteinase K to digest proteins. The DNA was purified with the DNA purification kit, and 50 μl TE Buffer was added to elution. 1 μl DNA sample was used for qPCR, and the primers used for qPCR are listed in Additional file 13: Table S5.

### Splicing efficiency of FLC measurement

The splicing efficiency of FLC was determined as previously [44]. Briefly, 5 μg of total RNA was reverse-transcribed into cDNA, the cDNA was used as template in qPCR to amplify FLC intron 1 spliced with primer F1 and R1, which cover the splicing junction; FLC intron 6 spliced with primer F6 and R6, which cover the splicing junction; FLC intron 1 unspliced with primer F1 and R1’, FLC intron 6 unspliced with primer F6’ and R6. Splicing efficiency was calculated by the level of spliced RNA normalized to the level of unspliced RNA.

### RNA-seq analyses

Seven-day-old Col-0 and *fer-4* seedlings grown under LD conditions and collected at ZT 4 were used for total RNA extraction. Total RNA was extracted with the mirVana miRNA Isolation Kit (Ambion, AM1561) following the manufacturer’s protocol. RNA integrity was evaluated using the Agilent 2100 Bioanalyzer (Agilent Technologies, Santa Clara, CA, USA). The samples with an RNA Integrity Number (RIN) ≥7 were subjected to the subsequent analysis. The libraries were constructed using the TruSeq Stranded mRNA LT Sample Prep Kit (Illumina, San Diego, CA, USA) according to the manufacturer’s instructions. Illumina sequencing was performed in Shanghai OE Biotech. Co., Ltd. with the Illumina sequencing platform (Illumina HiSeq X Ten).

Raw data (raw reads) were processed by using the NGS QC Toolkit [56]. The low quality reads were removed. The remaining reads were mapped to the *Arabidopsis* TAIR10 genome by using hisat2 [57]. Splicing events were identified by rMATS, and classification was obtained according to Matlin et al. [58, 59]. *P* value < 0.05 and inclusion level difference > 0.05 or < − 0.05 were set as the threshold for significant differential splicing changes. The FPKM value of each gene was calculated using cufflinks [60], and the read counts of each gene were obtained by htseq-count [61]. Differentially expressed genes (DEGs) were identified using the DESeq (2012) functions estimateSizeFactors and nbinomTest. *P* value < 0.05 and fold change > 2 or fold change < 0.5 were set as the threshold for significantly differential expression. Hierarchical cluster analysis of DEGs was performed to explore gene expression patterns. The raw RNA-seq data were uploaded to the NCBI database with the accession number SRX5988587.

### Statistics

Any significant differences in data were analyzed by Student’s t-test or by multivariate comparison (one-way ANOVA) using SPSS (version 17.0) software. All statistical tests were clearly described in the figure legends and/or in the methods section.

## Supplementary information

**Additional file 1: Figure S1.** Expression levels of *FER* under LD, SD and LL. conditions using the web-based tool *Diurnal* Expression of *FER* was associated with the *Arabidopsis* Columbia strain 0 grown under SD circadian conditions. Expression of *FER* under LD conditions was associated with *Arabidopsis* Ler grown under LD circadian conditions. Expression of *FER* under LL conditions was associated with *Arabidopsis* Col-0 grown under the circadian conditions of light (12 h, 12 h, and 24 h).

**Additional file 2: Figure S2.** Chromatin of several *CrRLK1L* subfamily genes containing the EE motif (A) The CCA1-bound EE motifs. (B) Sequences containing the EE motif in the chromatin of several *CrRLK1L* subfamily genes. The number indicates the length of the sequence starting upstream of the ATG start codon.

**Additional file 3: Figure S3.** *fer-4* mutant alters the amplitudes of certain clock genes (E) qPCR analysis of *CCA1* (A), *LHY* (B), *TOC1* (C) and *PRR7* (D) expression levels in WT and *fer-4* mutant plants under LD. The 7-day-old seedlings were harvested at 3-h intervals. Day and night are denoted by white and black bars, respectively. The amplitude was analyzed using BioDare2. All experiments were performed at least three times with similar results, and the error bars indicate the SD of three technical replicates (*P < 0.05, **P < 0.01, Student’s t-test).

**Additional file 4: Figure S4.** Loss of FER delays flowering in *Arabidopsis* (A) The flowering genotypes of the WT (C24) and *srn* mutant (another FER null mutant) under LD conditions. The plants were grown in soil under light conditions at an intensity of 50 μmol m-2 S-1 for 45 d. A representative experiment of three independent replicates is shown. (B) The flowering times measured as days to flower under LD conditions. Values are the mean ± SD of at least 15 plants. The asterisk indicates a significant difference (**P < 0.01, one-way ANOVA with Tukey’s test). (C) Number of rosette leaves in WT (*n* = 15) and *srn* (*n* = 15) under LD conditions. The bar indicates the SD (**P < 0.01, one-way ANOVA with Tukey’s test).

**Additional file 5: Figure S5.** The relative mRNA levels of unspliced FLC intron 1 and intron 6 decreased in *fer-4* mutant. Total RNA were extracted from 7-d-old seedling grown under LD condition and collected at ZT 12. The primer pairs F1/R1’ and F6’/R6 were used to detect the unspliced RNA for FLC introns 1 and 6, respectively. Primer pairs F1/R1 and F6/R6 were used to detect the spliced mRNA. The experiments were performed three times the bar indicates the mean ± SD (**P < 0.01, Student’s t-test).

**Additional file 6: Figure S6.** Quality analyses of RNA-seq data from wild-type and *fer-4* mutant (A) Mapping results of RNA reads. (B) Distribution of RNA-seq read coverage in the Col-0 and *fer-4* mutant were plotted along the length of the transcriptional unit. The X-axis indicates the percentile of the gene body, and the y-axis shows the read number. (C) Distribution of the RNA-seq reads along annotated *Arabidopsis* genomic features in Col-0 and *fer-4.* Among the mapped reads, more than 99% of reads map to the annotated exon. (D) Summary of genes whose transcripts were upregulated or downregulated in the *fer-4* mutant as determined by RNA-seq experiments.

**Additional file 7: Figure S7.** qPCR analysis of RALF1 mRNA levels in different tissues RALF1 (A), RALF23 (B) expression patterns as illustrated from the bar website: http://bar.utoronto.ca/. (C) qPCR analysis of RALF mRNA showed that RALF1 is highly expressed in root, and also expressed in shoot apex. RNA were extracted from 10-day-old root, shoot apex and leaves. The expression of RALF1 in leaves is lower compare to other two tissue types. *ACTIN2* was used as an internal control.

**Additional file 8: Figure S8**. Isolation and characterization of the *ralf1* mutant and *RALF1-OX* lines **(A)** Verification of the location of the T-DNA insertion described in SIGnAL (http://signal.salk.edu/cgi-bin/tdnaexpress). For the ATG start codon, the black boxes are exons, and the white boxes are the UTR. The exact sites of the T-DNA insertions (indicated by triangles) were mapped by PCR and DNA sequencing of the PCR products. **(B)** The T-DNA insert was present in the *ralf1* mutant but not in the WT genomic DNA. **(C)** The relative mRNA levels of the *RALF1* genes in the WT and eight different *RALF1-OX* lines. *ACTIN2* was used as the internal control to calculate the relative mRNA levels. The experiments were performed at least three times with similar results. **(D)** Transgenic *RALF1-OX* lines were verified by PCR. WT plants were used as a negative control.

**Additional file 9: Table S1.** Genes with significant (> 2-fold, *p* < 0.05) expression in *fer-4* compare with WT as determined by RNA-seq anaylsis.

**Additional file 10: Table S2.** Genes with alternative splicing events in *fer-4* plant as determined by RNA-seq analysis.

**Additional file 11: Table S3.** Enrichment analysis showing the enriched categories for*FER* knockout compared with WT.

**Additional file 12: Table S4.** Flowering relate genes with alternative splicing events in *fer-4* plant as determined by RNA-seq analysis.

**Additional file 13: Table S5.** Primers list.

## Data Availability

All data generated or analysed during this study are included in this published article and its supplementary information files.

## Abbreviations

AS: Alternative Splicing
CCA1: CIRCADIAN CLOCK-ASSOCIATED1
CCA1-OX: CCA1-overexpressing
CO: CONSTANS
DEGs: Differentially expressed genes
EC: evening complex
ELF3: EARLY FLOWERING 3
EMF2: EMBRYONIC FLOWER 2
FCA: FLOWERING CONTROL LOCUS A
FER: FERONIA
FLC: FLOWERING LOCUS C
FLD: FLOWERING LOCUS D
FLM: FLOWERING LOCUS M
FT: FLOWERING LOCUS T
GI: GIGANTEA
HERK1: HERCULES1
LD: Long day
LHY: LATE ELONGATED HYPOCOTYL
LL: Continuous light
LUX: LUX ARRHYTHMO
MAF: MADS AFFECTING FLOWERING
PP2A: Protein phosphatase 2A
PRMT5: Protein arginine methyltransferase 5
PRR7: PSEUDO-RESPONSE REGULATOR7
PRR9: PSEUDO-RESPONSE REGULATOR9
RALF: Rapid Alkalinization Factor
SC35: The 35-kDa splicing factor
SD: Short day
snRNPs: Small nuclear ribonucleoprotein particles
SOC1: SUPPRESSOR OF CONSTANS 1
SR: Serine/arginine-rich
SVP: SHORT VEGETATIVE PHASE
THE1: THESEUS 1
TOC1: TIMING OF CAB EXPRESSION 1
VRN1: VERNALIZATION 1
ZT: Zeitgeber time
