# Supplementary figures and images for: Receptor kinase FERONIA regulates flowering time in Arabidopsis
Source: BMC Plant Biol. 2020 Jan 16;20:26. doi: 10.1186/s12870-019-2223-y (PMC6966814; doi:10.1186/s12870-019-2223-y)

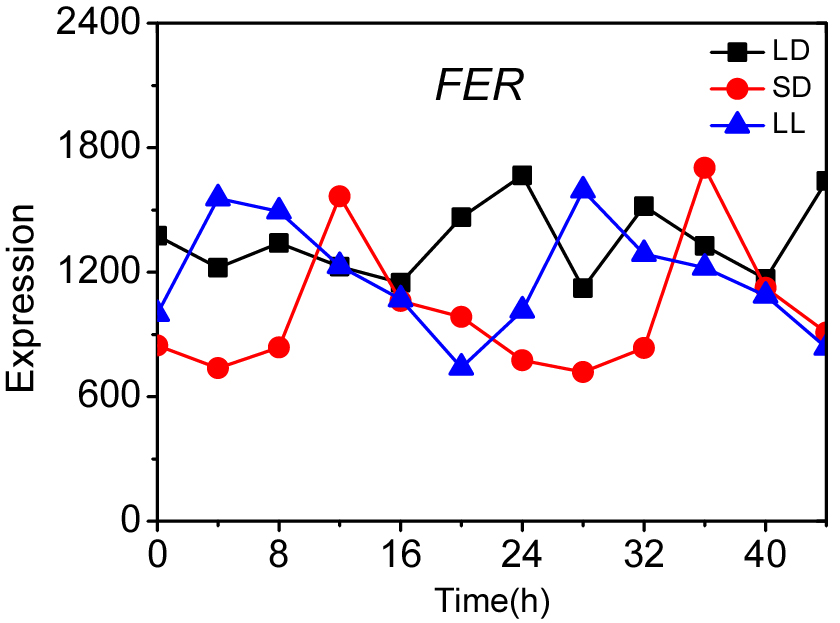

Supplement: Supplementary file 1 — Additional file 1: Figure S1. Expression levels of FER under LD, SD and LL. conditions using the web-based tool Diurnal Expression of FER was associated with the Arabidopsis Columbia strain 0 grown under SD circadian conditions. Expression of FER under LD conditions was associated with Arabidopsis Ler grown under LD circadian conditions. Expression of FER under LL conditions was associated with Arabidopsis Col-0 grown under the circadian conditions of light (12 h, 12 h, and 24 h). [file 12870_2019_2223_MOESM1_ESM.jpg]

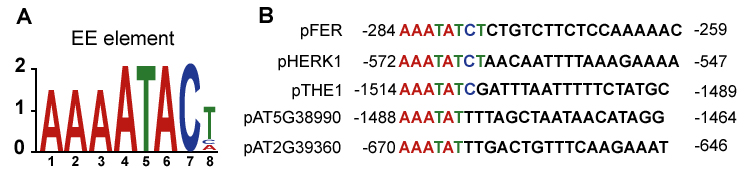

Supplement: Supplementary file 2 — Additional file 2: Figure S2. Chromatin of several CrRLK1L subfamily genes containing the EE motif (A) The CCA1-bound EE motifs. (B) Sequences containing the EE motif in the chromatin of several CrRLK1L subfamily genes. The number indicates the length of the sequence starting upstream of the ATG start codon. [file 12870_2019_2223_MOESM2_ESM.jpg]

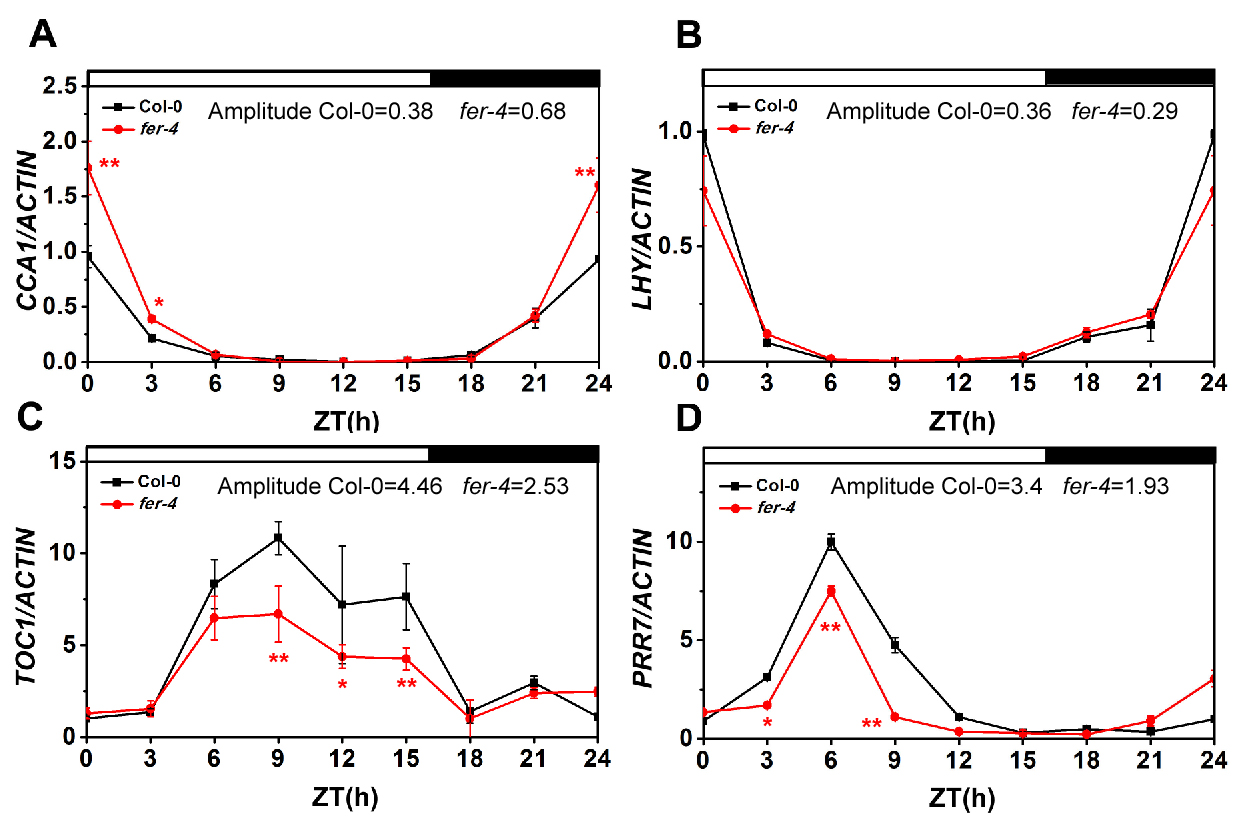

Supplement: Supplementary file 3 — Additional file 3: Figure S3. fer-4 mutant alters the amplitudes of certain clock genes (E) qPCR analysis of CCA1 (A), LHY (B), TOC1 (C) and PRR7 (D) expression levels in WT and fer-4 mutant plants under LD. The 7-day-old seedlings were harvested at 3-h intervals. Day and night are denoted by white and black bars, respectively. The amplitude was analyzed using BioDare2. All experiments were performed at least three times with similar results, and the error bars indicate the SD of three technical replicates (*P < 0.05, **P < 0.01, Student’s t-test). [file 12870_2019_2223_MOESM3_ESM.jpg]

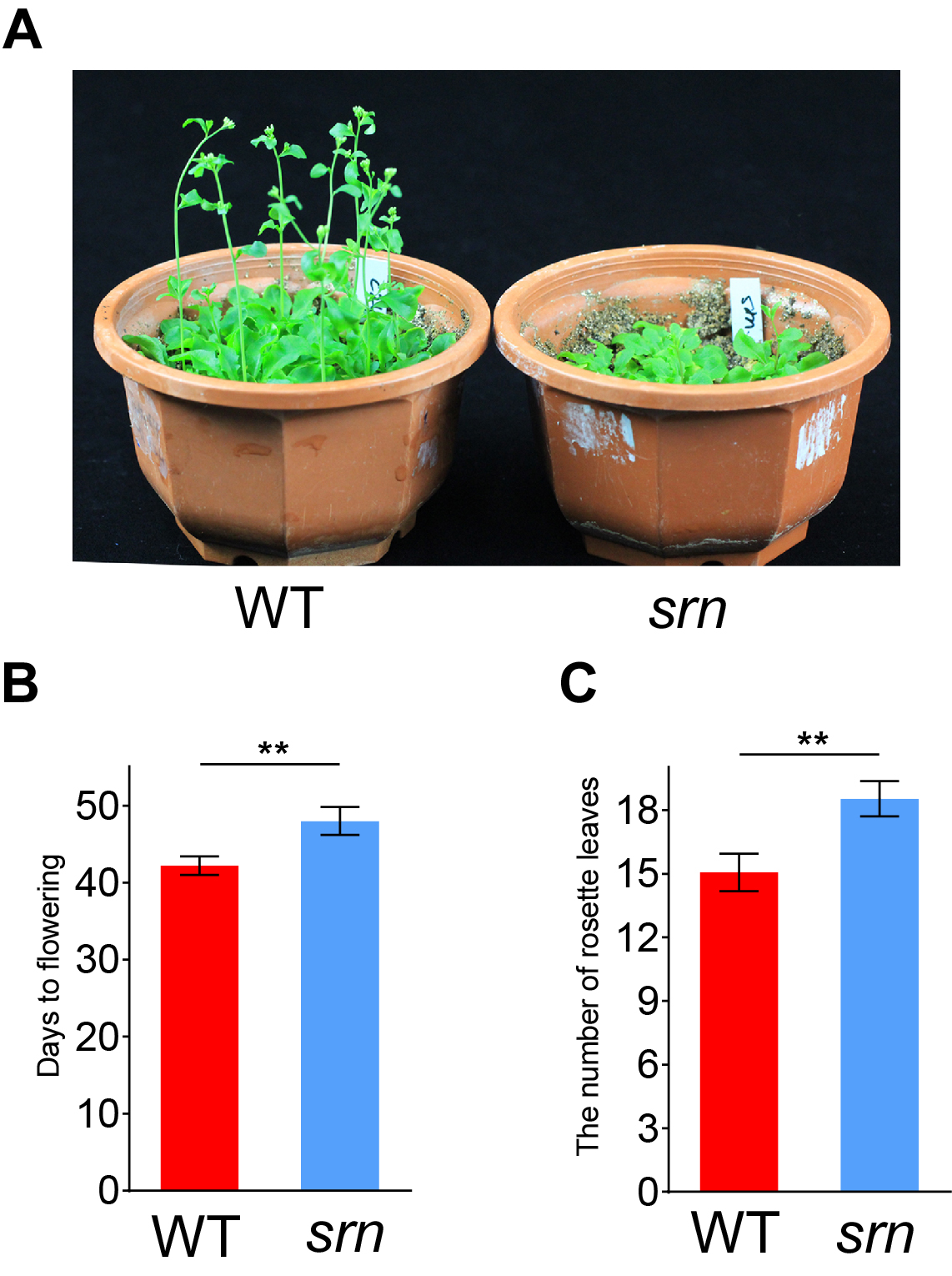

Supplement: Supplementary file 4 — Additional file 4: Figure S4. Loss of FER delays flowering in Arabidopsis (A) The flowering genotypes of the WT (C24) and srn mutant (another FER null mutant) under LD conditions. The plants were grown in soil under light conditions at an intensity of 50 μmol m-2 S-1 for 45 d. A representative experiment of three independent replicates is shown. (B) The flowering times measured as days to flower under LD conditions. Values are the mean ± SD of at least 15 plants. The asterisk indicates a significant difference (**P < 0.01, one-way ANOVA with Tukey’s test). (C) Number of rosette leaves in WT (n = 15) and srn (n = 15) under LD conditions. The bar indicates the SD (**P < 0.01, one-way ANOVA with Tukey’s test). [file 12870_2019_2223_MOESM4_ESM.jpg]

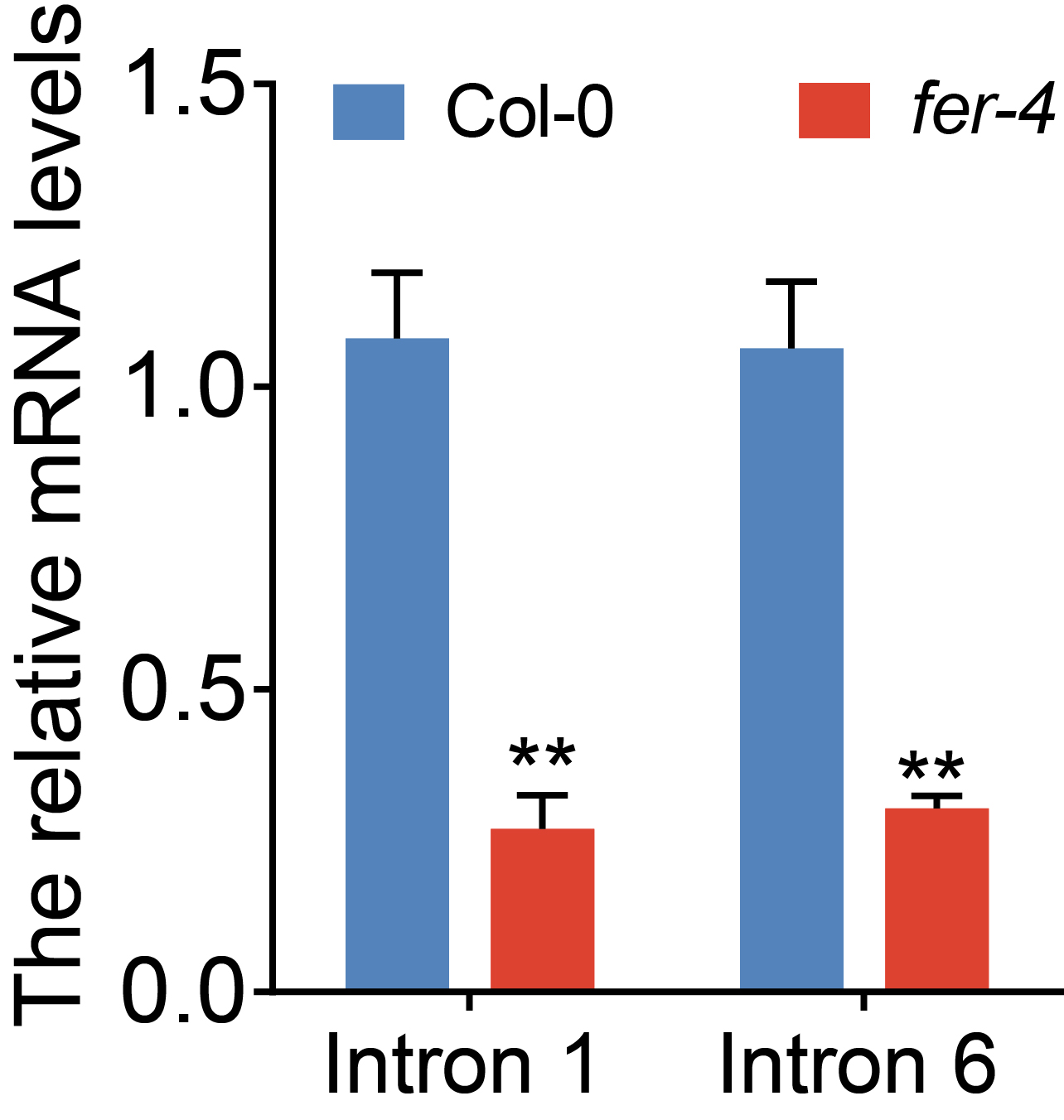

Supplement: Supplementary file 5 — Additional file 5: Figure S5. The relative mRNA levels of unspliced FLC intron 1 and intron 6 decreased in fer-4 mutant. Total RNA were extracted from 7-d-old seedling grown under LD condition and collected at ZT 12. The primer pairs F1/R1’ and F6’/R6 were used to detect the unspliced RNA for FLC introns 1 and 6, respectively. Primer pairs F1/R1 and F6/R6 were used to detect the spliced mRNA. The experiments were performed three times the bar indicates the mean ± SD (**P < 0.01, Student’s t-test). [file 12870_2019_2223_MOESM5_ESM.jpg]

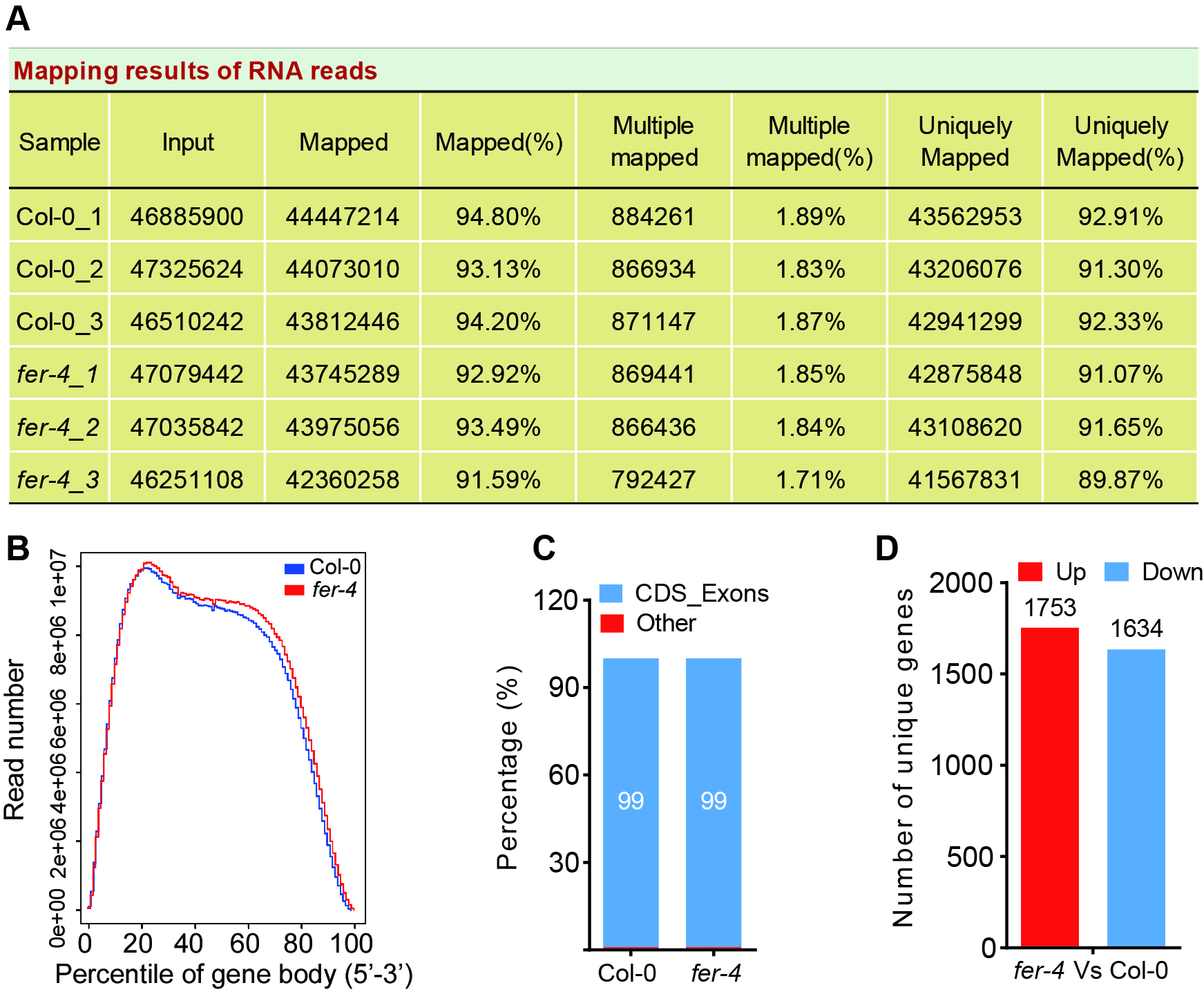

Supplement: Supplementary file 6 — Additional file 6: Figure S6. Quality analyses of RNA-seq data from wild-type and fer-4 mutant (A) Mapping results of RNA reads. (B) Distribution of RNA-seq read coverage in the Col-0 and fer-4 mutant were plotted along the length of the transcriptional unit. The X-axis indicates the percentile of the gene body, and the y-axis shows the read number. (C) Distribution of the RNA-seq reads along annotated Arabidopsis genomic features in Col-0 and fer-4. Among the mapped reads, more than 99% of reads map to the annotated exon. (D) Summary of genes whose transcripts were upregulated or downregulated in the fer-4 mutant as determined by RNA-seq experiments. [file 12870_2019_2223_MOESM6_ESM.jpg]

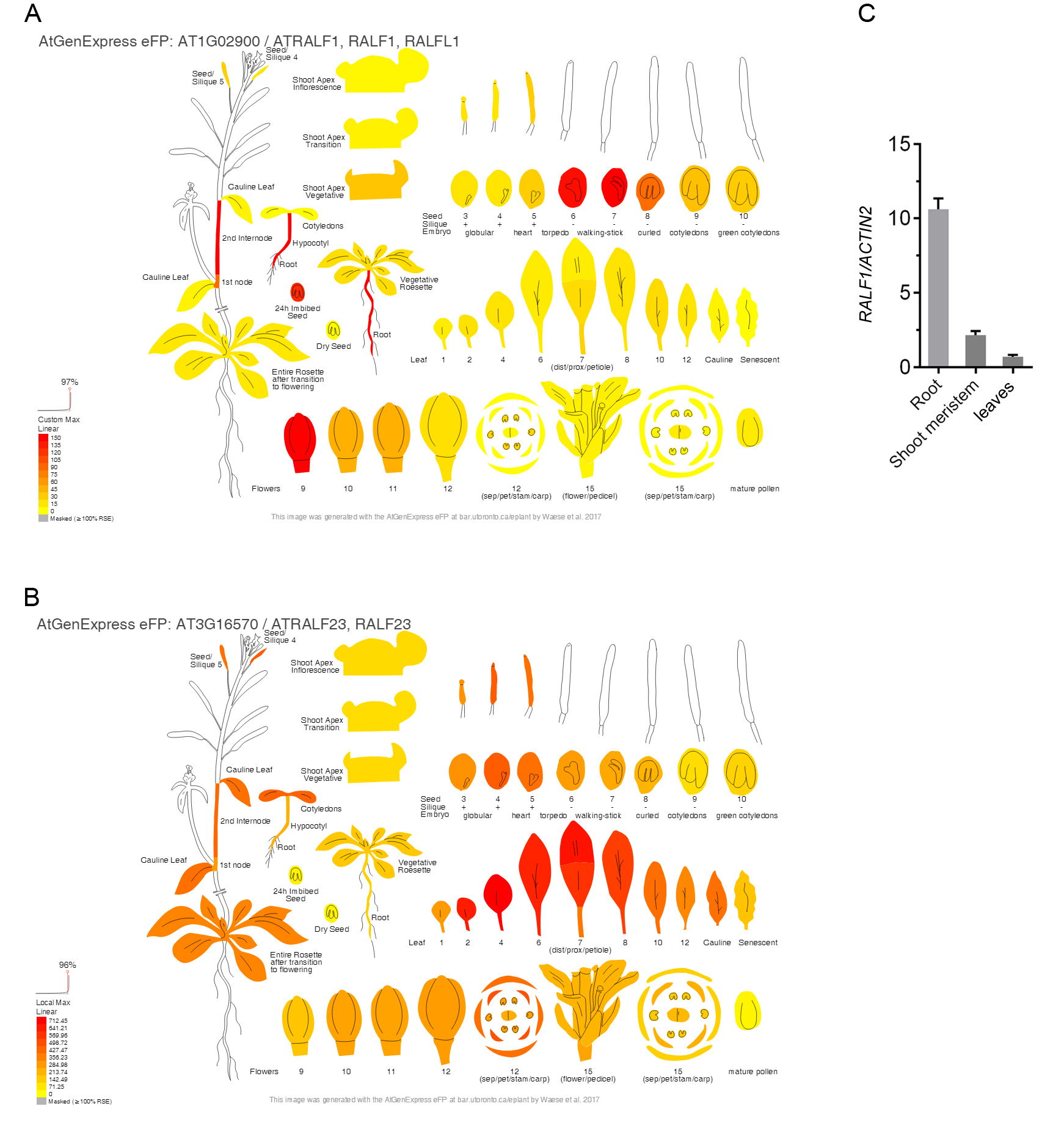

Supplement: Supplementary file 7 — Additional file 7: Figure S7. qPCR analysis of RALF1 mRNA levels in different tissues RALF1 (A), RALF23 (B) expression patterns as illustrated from the bar website: http://bar.utoronto.ca/. (C) qPCR analysis of RALF mRNA showed that RALF1 is highly expressed in root, and also expressed in shoot apex. RNA were extracted from 10-day-old root, shoot apex and leaves. The expression of RALF1 in leaves is lower compare to other two tissue types. ACTIN2 was used as an internal control. [file 12870_2019_2223_MOESM7_ESM.jpg]

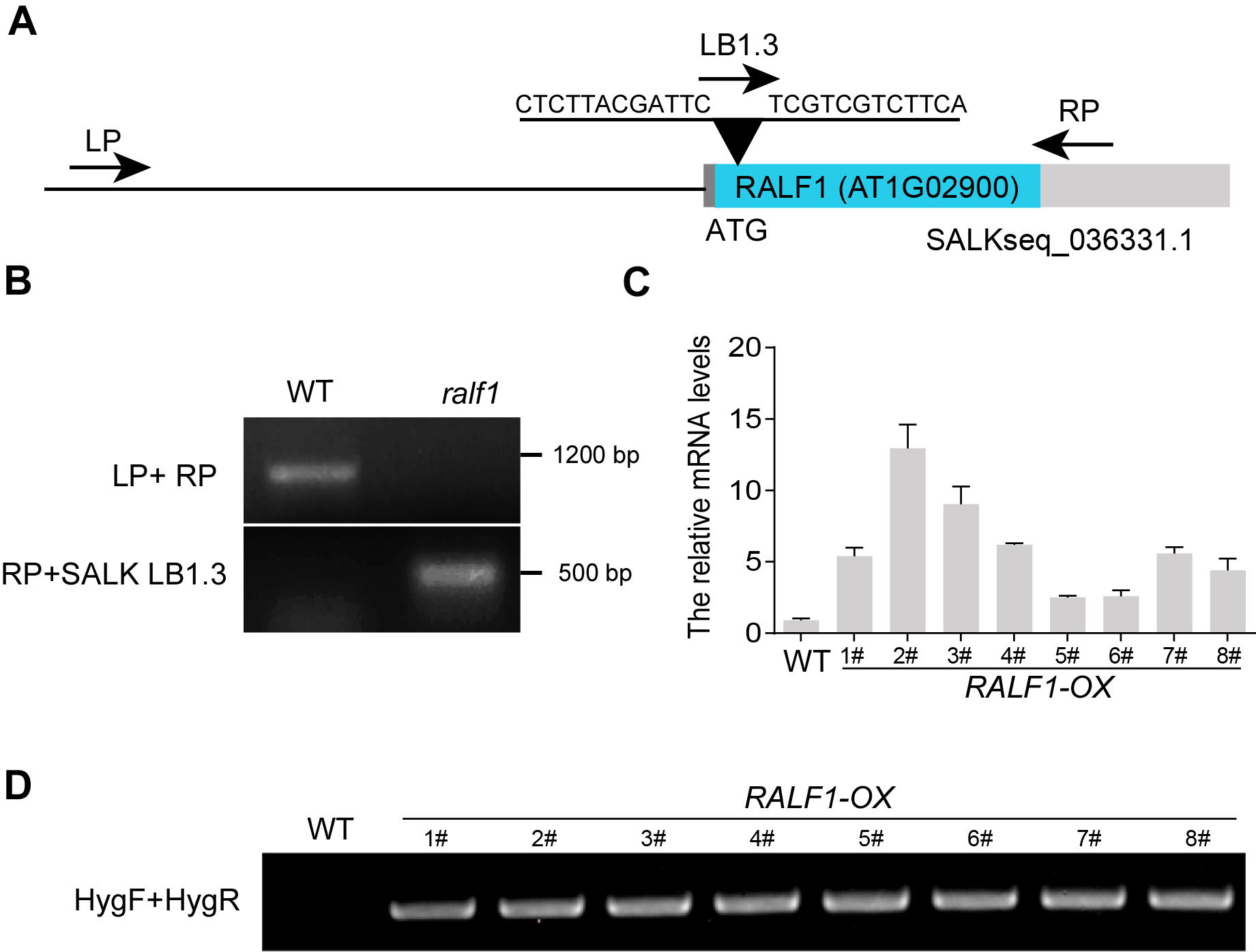

Supplement: Supplementary file 8 — Additional file 8: Figure S8. Isolation and characterization of the ralf1 mutant and RALF1-OX lines (A) Verification of the location of the T-DNA insertion described in SIGnAL (http://signal.salk.edu/cgi-bin/tdnaexpress). For the ATG start codon, the black boxes are exons, and the white boxes are the UTR. The exact sites of the T-DNA insertions (indicated by triangles) were mapped by PCR and DNA sequencing of the PCR products. (B) The T-DNA insert was present in the ralf1 mutant but not in the WT genomic DNA. (C) The relative mRNA levels of the RALF1 genes in the WT and eight different RALF1-OX lines. ACTIN2 was used as the internal control to calculate the relative mRNA levels. The experiments were performed at least three times with similar results. (D) Transgenic RALF1-OX lines were verified by PCR. WT plants were used as a negative control. [file 12870_2019_2223_MOESM8_ESM.jpg]
